# Unequal distributional change in body mass index among pre-pregnant women and their male partners in northern Sweden: a quantile regression analysis

**DOI:** 10.1016/j.ssmph.2025.101877

**Published:** 2025-11-06

**Authors:** Fethi Mohammed Yusuf, Anni-Maria Pulkki-Brännström, Per E. Gustafsson, Anneli Ivarsson, Marie Lindkvist, Masoud Vaezghasemi

**Affiliations:** Department of Epidemiology and Global Health, Umeå University, SE-901 87, Umeå, Sweden

**Keywords:** BMI distribution, Education, Gender, Quantile regression, Sweden

## Abstract

**Background:**

Obesity is a global public health issue with increasing prevalence and notable differences across population. Previous studies on body mass index (BMI) trends and inequalities have focused on overweight/obesity prevalence or average BMI changes, overlooking differences across the BMI distribution. This study investigates whether changes in BMI distribution are uniform or different over time and educational attainment.

**Methods:**

This study is based on repeated cross-sectional surveys in Västerbotten, Sweden. Study participants were expectant parents visiting antenatal care (2010–2019) as part of the Salut Programme. During early pregnancy, 18,215 women and 17,890 male partners completed questionnaires. Quantile regression analyses were conducted to assess BMI distribution changes over time and by education for men and women.

**Results:**

The BMI distribution for women showed a sharper increase in the upper tail in 2018/19 compared to 2010/11, whereas for men, the upper tail showed a gradual rise over years. Similar changes in BMI distributions were observed over time across both educational groups, with a notable increase in the higher BMI segments.

**Conclusion:**

The study revealed weight gain inequalities, with higher BMI segments experiencing a disproportionately higher rise compared to others. Identifying high-risk groups in vulnerable settings will better equip decision-makers to design and implement targeted intervention strategies to reduce overweight and obesity.

## Highlights

• Weight gain was uneven; higher BMI groups experienced greater increases over time.
• BMI shifted upward across all sex and education groups.
• Education did not consistently protect against rising BMI levels.
• Interventions should target higher BMI groups to reduce obesity.

## 1 Introduction

Overweight and obesity are global health concerns that are increasing worldwide (Finucane et al., 2011). The global prevalence of obesity more than doubled between 1990 and 2022 (Brauer et al., 2024; WHO, 2025). Sweden faces the same growing challenge, with adult overweight and obesity dramatically increasing during the last decades (Folkhälsomyndigheten, 2024; Vogt et al., 2024). Concurrently, the proportion of individuals with normal weight has decreased, indicating a shift towards higher weight categories.

Moreover, a recent report from Sweden highlights significant disparities in the population (Folkhälsomyndigheten, 2024) supported by several studies demonstrating a social gradient in obesity (Eek & Östergren, 2009; Johansson, 2010; Yusuf et al., 2023). Education contributes to these inequalities, with overweight being more prevalent among those with lower education (Bjermo et al., 2015; Folkhälsomyndigheten, 2024; Molarius et al., 2016). For example, among pregnant women in Sweden, the prevalence of overweight/obesity is 54 % for those with secondary education and 40 % for those with post-secondary education (Folkhälsomyndigheten, 2025). There are also gender differences in the prevalence of overweight and obesity, with higher overall prevalence among women globally (WHO, 2025). However, in high income nations, being overweight is more common among men, and studies in Sweden show similar patterns (Hemmingsson et al., 2021; Yusuf et al., 2023).

These differences in overweight or obesity prevalence between men and women and among different educational levels underscore the importance of examining BMI distribution beyond simple averages or prevalence rates. While prior research in Sweden has primarily focused on mean BMI or the overweight and obesity prevalence, such approaches may potentially obscure important variations across the BMI distribution. Identifying sub-groups with the highest BMI, or the fastest increase is crucial for effective public health strategies. Rose's concept of population-based prevention, introduced in the 1970s and 1980s, was developed at a time when risk prediction relied on just one or two factors and had limited discriminative power (Rodgers et al., 2004; Subramanian et al., 2018; Zulman et al., 2008). In contrast, modern risk prediction tools can more accurately capture heterogeneity in treatment benefits across populations (Hayward et al., 2005).

By examining changes across the entire BMI distribution, we can gain a more comprehensive understanding of who is gaining more weight overtime, including differences between genders. Furthermore, analysing how BMI distribution changes differ across educational levels enables us to identify specific patterns and disparities between these groups. Despite this need, no study has specifically analysed differential change in the entire distribution of BMI in Sweden. Therefore, this study aimed (i) to examine if BMI is changing equally across all segments of the BMI distribution among pre-pregnant women and their partners in Västerbotten from 2010 to 2019, and (ii) to observe differences between low and high education groups.

## 2 Methods

### 2.1 Study design and study population

This study used data from a repeated cross-sectional study collected in Antenatal Care as part of the Salut Programme, an ongoing universal health promotion programme in the county of Västerbotten, northern Sweden (Eurenius et al., 2011; Pulkki-Brännström et al., 2020). Pregnant women and their partners were identified as the target population; and they were invited to fill out a paper questionnaire that included items related to health, lifestyle, educational attainment, occupation, family situation, and living conditions. The present study is based on questionnaire data gathered between 2010 and 2019.

The study sample consisted of pregnant women and male partners who completed a questionnaire before their first routine visit to Antenatal Care, around the 11th week of gestation. Between 2010 and 2019, a total of 42,249 questionnaires were filled out by the study participants. Among the participants, 3575 observations were excluded due to missing values on BMI, age or education. In addition, 2566 did not consent to research, and an additional three participants were excluded because their reported BMI exceeded 60. Thus, the BMI ranged from 12 kg/m^2^ to 60 kg/m^2^. These exclusions contributed to the observed differences in the number of male and female respondents presented in Table 1. Therefore, the final sample size included a total of 36,105 participants: 18,215 pregnant women and 17,890 male partners. During the study period a total of 29,047 live births were recorded in Västerbotten (Vogt et al., 2024), which indicate that the final study's sample comprised approximately 63 % of all women with a pregnancy resulting in a live birth in Västerbotten during the study period. This proportion reflects population-level coverage rather than a response rate from a sampled survey. Exclusions were primarily due to missing data or lack of consent, with no evidence of systematic bias (e.g., missingness was not associated with low education), suggesting that the risk of selection bias is minimal (analysis not shown). Some women may have received antenatal care outside the region, moved during pregnancy, or delivered twins, which also contributed to the lower number of women in the final sample in comparison to total live births during the study period.

**Table 1:** Characteristics of the sample (n = 36,105) in Västerbotten from 2010 to 2019.

Women							Men					
Year	Total (N)	Age	BMI			Education	Total (N)	Age	BMI			Education
		Mean (SD)	Mean (SD)	Median	Skewness	Low		Mean (SD)	Mean (SD)	Median	Skewness	Low
2010	1682	29.8 (5)	23.9 (4.4)	22.9	1.3	666 (40 %)	1504	32.2 (5.8)	25.7 (3.4)	25.3	1.3	820 (55 %)
2011	2019	29.9 (5)	23.6 (4.4)	22.6	1.6	783 (39 %)	1956	32.1 (5.9)	25.7 (3.5)	25.2	1.1	1029 (53 %)
2012	1989	29.7 (4.9)	23.8 (4.5)	22.7	1.5	740 (37 %)	2111	32.3 (5.7)	25.8 (3.6)	25.3	0.9	1113 (53 %)
2013	1683	29.7 (4.9)	23.7 (4.3)	22.8	1.4	630 (37 %)	1619	32.2 (5.8)	25.7 (3.6)	25.2	1.6	852 (53 %)
2014	1286	29.8 (4.7)	23.8 (4.2)	23.1	1.3	452 (35 %)	1247	31.9 (5.5)	25.7 (3.4)	25.3	0.8	640 (51 %)
2015	1867	29.8 (4.8)	23.9 (4.5)	22.9	1.6	689 (37 %)	1869	32.1 (5.7)	25.8 (3.8)	25.3	1.5	985 (53 %)
2016	2034	30.1 (4.7)	23.9 (4.3)	22.9	1.5	738 (36 %)	1952	32.3 (5.7)	25.8 (3.8)	25.2	1.4	1000 (51 %)
2017	1843	30.1 (4.6)	23.9 (4.4)	22.9	1.6	644 (35 %)	1864	32.3 (5.5)	26.1 (3.9)	25.5	1.7	978 (53 %)
2018	1976	30.0 (4.6)	24.3 (4.7)	23.2	1.6	717 (36 %)	2002	32.2 (5.6)	26.0 (4.1)	25.4	1.4	1063 (53 %)
2019	1836	30.5 (4.4)	24.3 (4.5)	22.9	1.2	603 (33 %)	1766	32.7 (5.5)	26.1 (4.0)	25.4	1.5	861 (49 %)

### 2.2 Measures

BMI was based on self-reported weight and height with weight (kg) divided by height squared (m^2^). For men, BMI was determined as the current BMI when filling out the questionnaire. However, for women, it was based on reported weight just before the pregnancy. Age-adjusted BMI was calculated separately for women and men, in order to account for differing patterns in the relationship between BMI and age over time in each group. It was obtained for women by regressing BMI on age and on a quadratic term for age, and by adding residuals of the linear model to the women's grand mean of BMI (Razak et al., 2013; Vaezghasemi et al., 2016). For male partners, the procedure was similar but no quadratic term for age was used in the linear regression model. For men, the relationship between age and BMI appeared linear, and adding a quadratic term did not enhance model performance. To maintain parsimony and avoid overfitting, we excluded the quadratic term from the model for men.

Calendar years ranged from 2010 to 2019 and were recoded as biennial intervals (i.e. every two years). This is helpful for clearly visualizing the evolution of BMI through graphical illustrations, as using annual intervals would make the visuals too busy and obscure the main trends in the data. After this, we referred to each biennial interval as a time period throughout the text. Education was recorded as the highest level of completed education in five levels, then dichotomized into low and high categories. The low category included (i) less than 9 years of schooling, (ii) compulsory school or the equivalent of 9 years of schooling, (iii) upper secondary school (high school) or the equivalent of 12 years of schooling. The high category included (iv) post-secondary education, less than 3 years, or (v) post-secondary education, 3 years or more.

### 2.3 Statistical analysis

Descriptive statistical analysis was done to summarise the characteristics of study participants. Numbers, percentages, median, mean, skewness and standard deviation are displayed in Table 1 for age, BMI and educational attainment by year for both genders. In addition, quantile regression analyses were used as the main analytical method. Quantile regression allows for the estimation of changes across the entire BMI distribution (Gebremariam et al., 2018), providing estimates (coefficients) at specific quantiles such as the 0.05, 0.25, 0.50, 0.75, and 0.95 quantiles (Bann et al., 2020). The quantile regression gives a coefficient estimate, standard error, and P value for each model covariate at each specified quantile. This contrasts with linear regression or logistic regression, in which we compare mean differences in BMI or proportion difference in overweight/obesity. Adopting this approach is important because what determines BMI in the left and the right tail (where health risks are higher) may be significantly different from what determines BMI in the centre of the distribution.

Therefore, quantile regression was used in this study to assess changes in BMI quantiles between different years for the study period 2010 to 2019. For the first aim, the changes were illustrated by different plots, where BMI difference based on quantile regression coefficients was plotted by quantile from 0.01 to 0.98 for each time period (2012/13–2018/19) against baseline time period (2010/11), separately for women and men. The 0.99 quantile was not plotted because the values were outside the scale range (>2). We selected 2 as the upper limit of the y-axis to ensure consistency across all graphs and to maintain a fixed scale. This approach allows for easier visual comparison between paired graphs presented side by side, enhancing interpretability for the reader. However, to ensure transparency and completeness, we have included all quantile estimates—including the 0.99 quantile—in the supplementary tables. Each line on the graph shows the estimated coefficient of the BMI distribution for its corresponding time period. If the two distributions being compared are the same, the dots lie on the line of equality, a horizontal line at 0.0. Dots above the line of equality represent a higher level of BMI at those quantiles compared to the reference time period (2010/11). In addition, if BMI is increasing uniformly, we would expect a parallel line above the horizontal line.

To address the second aim, we plotted the corresponding changes over time for each BMI quantile but stratified for education level in addition to gender. We also plotted the regression lines (slopes) over the study period at the 0.2, 0.4, 0.6, 0.8, and 0.99 BMI quantiles for the whole sample, further stratified by gender. Quantiles below 0.2 were not included because the trends at the lower end of the BMI distribution were relatively flat, and we aimed to focus on the more pronounced changes observed in the upper quantiles.

Moreover, we did period-to-period analysis (compared each time period to the previous one) to observe how BMI gradually evolved and to determine if there were any statistically significant differences between adjacent time periods. For example, the BMI distribution during 2012/13 was compared to 2010/11, and 2014/15 to 2012/13, and so on. The corresponding graphs are attached as supplementary data, Figs. S1–S6. Supplementary Tables S1–S4 provide the estimated quantile regression coefficients for the 0.01 to 0.99 quantiles for both men and women, including analyses stratified by education level. A coefficient of 1 indicates that BMI increased by 1 unit compared to the reference group, suggesting a meaningful shift in body mass at that specific quantile (Rodriguez-Caro et al., 2016; Staffa et al., 2019). We have highlighted estimates with statistically significant confidence intervals (i.e., those not including zero) using a distinct colour (green) in the supplementary tables. This visual distinction allows readers to easily identify which quantile estimates are statistically significant. All analyses were performed using R version 4.4.0 (the R Foundation for Statistical Computing), with the significance level set at a p-value of less than 0.05.

## 3 Results

The mean age and mean BMI of women were consistently lower than their male partners. The median BMI was around 23 kg/m^2^ for women and 25 kg/m^2^ for men throughout most of the study period. BMI was positively skewed across the years, as expected—generally more so in women. Although the degree of skewness varied slightly over time, no clear trend was observed. About one third of the women and half of the men had lower than 12 years of schooling and educational level increased gradually over the entire study period (Table 1). In addition, Table 1 showed the proportions of participants in the high and low education groups remained relatively stable throughout the study period.

**Fig. 1:**
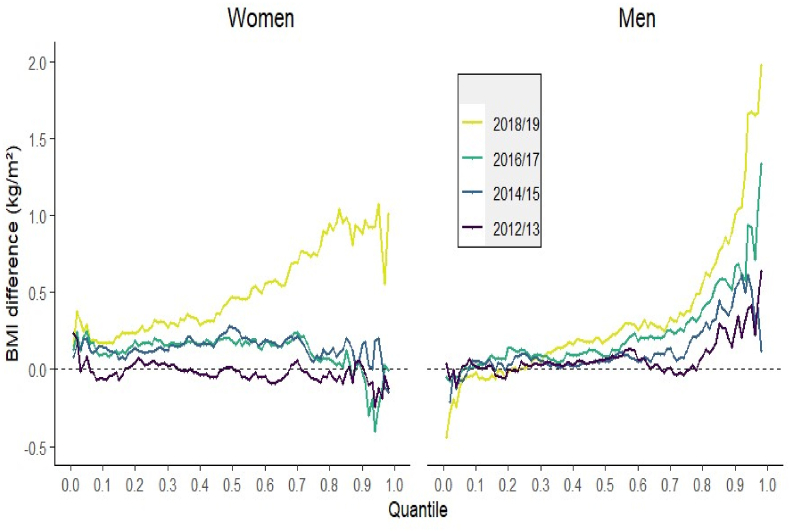
BMI differences for the 0.01 to 0.98 quantiles of the BMI distribution for women and men. A dot above the 0.0 line means that the predicted BMI value at this specific quantile of the BMI distribution is higher those time periods compared to 2010/11. A dot below the 0.0 line means the opposite.

Table 1 also suggests a slight rise in mean BMI (1.3 %) and standard deviation among women during last survey waves. This corresponds to a sudden increase in the BMI distribution among women during 2018/19, as shown in Fig. 1. Compared to 2010/11, BMI rose across nearly all segments in 2018/19 (see Supplementary Table S1). For instance, BMI increased by nearly 1 unit above the 0.8 quantile during this period. Additionally, a period-to-period comparison revealed statistically significant differences, particularly between 2016/17 and 2018/19. During this interval, BMI increased by 0.2–1.3 units above the 0.5 quantile, indicating a notable upward shift in the upper half of the distribution (see Supplementary Table S2). However, for the other time periods, changes in BMI did not appear to be statistically significant.

In contrast to the pattern observed among women, the BMI distribution among men showed a more gradual upward trend beginning in 2014/15, relative to the baseline year of 2010/11 (Fig. 1). This increase became particularly evident in the upper two-thirds of the distribution by 2018/19. Notably, significant gains were also detected in narrower segments during earlier periods—specifically above the 0.55 quantile in 2016/17 and above the 0.81 quantile in 2014/15 (Supplementary Table S3). At the 0.9 quantile, BMI increased by 0.5, 0.7, and 1 unit during 2014/15 and the subsequent time points compared to 2010/11, suggesting a consistent upward shift in the upper tail of the distribution over time. Despite these trends, period-to-period comparisons revealed that changes in BMI among men were not statistically significant across any segment of the distribution (Supplementary Table S4).

In contrast to the pattern observed among women, the BMI distribution among men showed a more gradual upward trend beginning in 2014/15, relative to the baseline year of 2010/11 (Fig. 1). This increase became particularly evident in the upper two-thirds of the distribution by 2018/19. Notably, significant gains were also detected in narrower segments during earlier periods—specifically above the 0.55 quantile in 2016/17 and above the 0.81 quantile in 2014/15 (Supplementary Table S3). At the 0.9 quantile, BMI increased by 0.5, 0.7, and 1 unit during 2014/15 and the subsequent time points compared to 2010/11, suggesting a consistent upward shift in the upper tail of the distribution over time. Despite these trends, period-to-period comparisons revealed that changes in BMI among men were not statistically significant across any segment of the distribution (Supplementary Table S4).

**Fig. 2:**
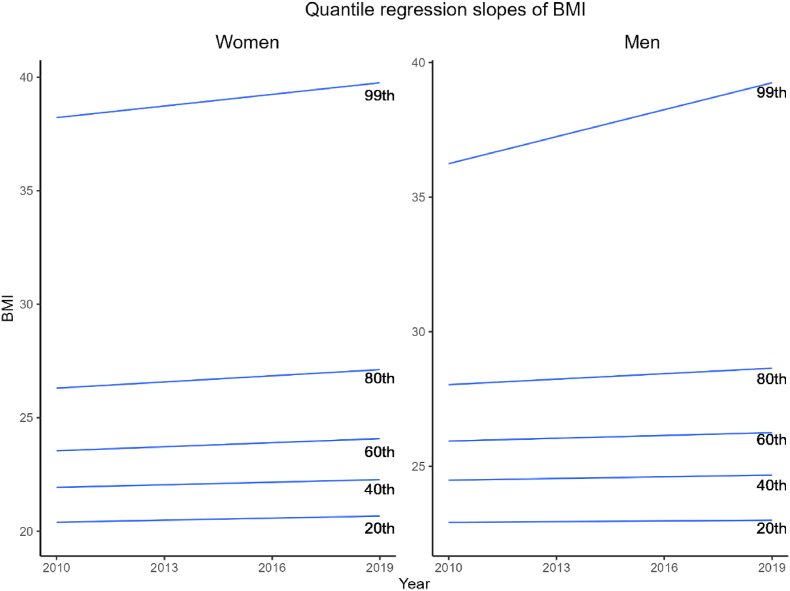
Regression slopes at the 0.2, 0.4, 0.6, 0.8 and 0.99 quantiles of BMI for women and men, 2010-2019.

Furthermore, Fig. 2 shows the regression slopes of BMI over time for both women and men, revealing distinct patterns across the distribution. The 0.99 quantile consistently increased over the entire duration for both genders, with consistently higher BMI observed in males. In contrast, the lower quantiles remained largely unchanged over time. This stability is particularly evident at the 0.2 quantile, where the regression line was nearly flat, indicating minimal variation across the years.

**Fig. 3:**
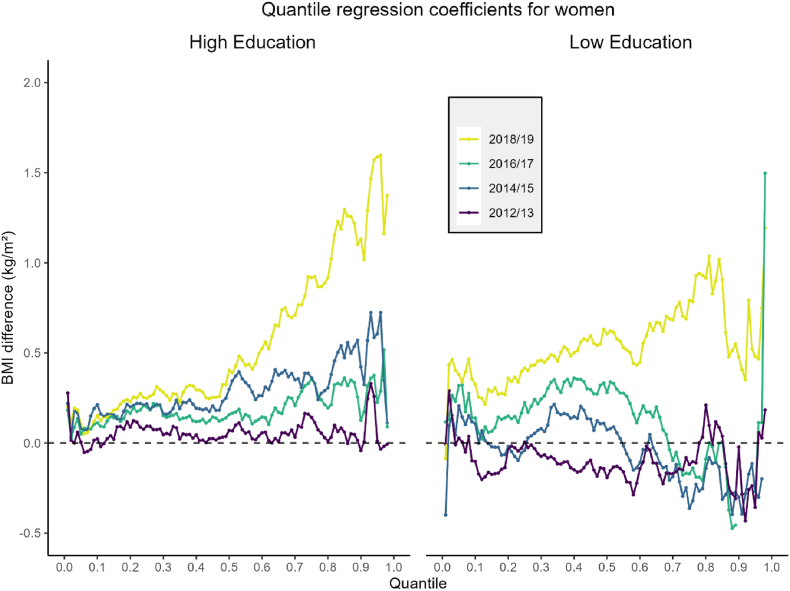
BMI differences for the 0.01 to 0.98 quantiles of the BMI distribution for women with both high and low educational attainment. Here, the line of equality at y = 0 corresponds to the reference time period (2010/2011).

With respect to educational attainment, we observed shifts in the BMI distribution among women and men over time too. Among women with high educational attainment, BMI increased significantly during both 2014/15 and 2018/19 compared to 2010/11 (Fig. 3). The 2018/19 period, in particular, was characterized by a marked rise across the distribution: BMI increased by 0.2 units at the 0.2 quantile and by as much as 1.5 units at the 0.95 quantile, reflecting a substantial widening of the distribution (Supplementary Table S1). Women with low educational attainment also experienced an upward trend in BMI during 2018/19, though the pattern was less pronounced.

**Fig. 4:**
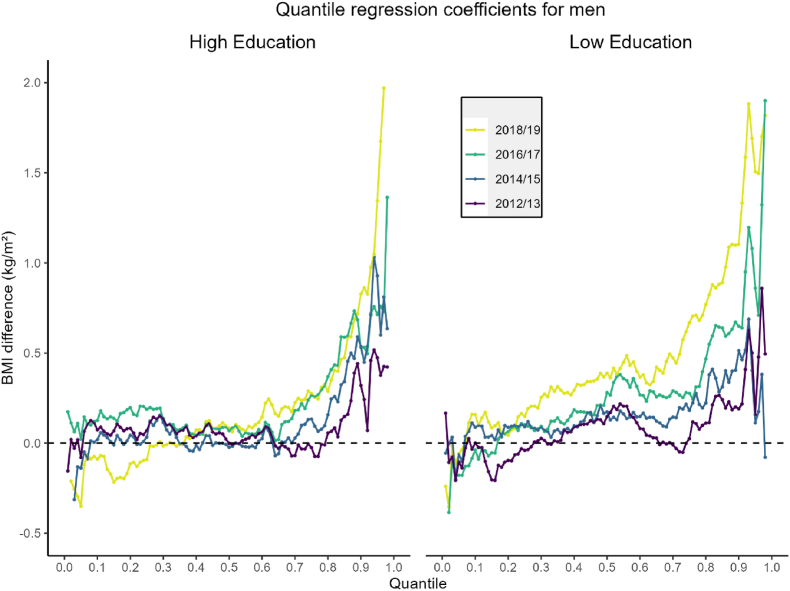
BMI differences for the 0.01 to 0.98 quantiles of the BMI distribution for men with both high and low educational attainment. Here, the line of equality at y = 0 corresponds to the reference time period (2010/2011).

In contrast, the trends among men varied slightly by educational level. Men with low educational attainment experienced more pronounced increases in BMI during both 2016/17 and 2018/19. This trend was especially evident in 2018/19, where statistically significant gains were observed across the upper two-thirds of the BMI distribution compared to 2010/11 (Fig. 4; Supplementary Table S3). However, among men with high educational attainment, significant increases were more limited, appearing only in the uppermost segments of the distribution—specifically above the 0.80 quantile—suggesting a more limited but still meaningful upward shift (Supplementary Table S3).

## 4 Discussion

This study presented evidence of varying temporal patterns in the entire distribution of BMI. One key finding is the unequal distribution of weight gain among the population, with those in higher BMI segments gaining weight faster than those in the lower segments. Our study also reveals a substantial gender difference in BMI changes over time, as well as differences related to educational attainment. Notably, those in higher BMI segments experienced increased weight gain later in the study period, regardless of educational level.

Our findings build on previous analysis of BMI trends in the same study population, which indicated an increase in the prevalence of overweight, accompanied by a more moderate decline in the prevalence of underweight (Vogt et al., 2024). The present paper, however, expands on this by demonstrating that the increases in BMI are not evenly distributed in the population. While the mean BMI for women remained relatively stable, except 2018/19 (Table 1), the increase was largely driven by weight gains in the upper segments of the BMI distribution (above the 0.50 quantile) (Supplementary Table S1).

On the other hand, weight gains for men were relatively stable across the BMI spectrum during the first half of the study period. However, during the second half, a small proportion of men at the higher end showed greater weight gains (Fig. S4). Moreover, our analysis revealed a sharpest increase in BMI at the 0.99 quantile for both genders, indicating a shift in BMI distribution towards higher values for both men and women. Our findings corroborate studies conducted in the USA (Krishna et al., 2015), Canada (Lebel et al., 2018) and Indonesia (Vaezghasemi et al., 2016) that examined BMI trends. These studies found asymmetric changes in BMI levels, with more significant changes occurring at the 95th percentile than at the 5th percentile among the adult population.

The disproportionately higher weight gain at the upper end of the BMI distribution is unlikely to be explained by cohort differences, as the age distribution remained stable over time and BMI was adjusted for age. Instead, this pattern may reflect gene–environment interactions, where individuals with genetic susceptibility to obesity are more affected by increasingly obesogenic environments—such as changes in diet, physical activity, and social norms surrounding weight (Albuquerque et al., 2017; Llewellyn, 2018).

Additionally, the greater weight gain among individuals in higher BMI segments may be driven by a combination of physiological, socioeconomic, and psychosocial factors (Swinburn & Egger, 2004). These factors are often exacerbated by the obesogenic environments of the modern world that make unhealthy choices easy and accessible. Although the body's metabolism naturally attempts to slow weight gain, factors such as reductions in physical activity –often due to mechanical problems associated with increased body weight– can counteract this mechanism, further accelerating weight gain in these individuals. Further qualitative research may help elucidate why and how disproportionate weight gain occurs among this group, including contextual, behavioral, and psychosocial factors that may not be captured through quantitative data.

Furthermore, we examined how BMI changes across different segments, stratified by education level. The increase in BMI during 2014/15 among women shown by deviation of line from line of equality depicted in Fig. S2 is driven by weight gain from previous period in the mid segment of BMI distribution. Similarly, the increase seen during the final period, 2018/19, was due to weight gain in the upper half of the BMI segments compared to from the preceding period (Supplementary Table S2). We also observed that the prevalence of overweight/obesity among highly educated women rose notably from 56 % to 60 % during the study period (data not shown). This finding is consistent with a recent Swedish report showing increased obesity among pregnant women across all educational groups since 2019 (Folkhälsomyndigheten, 2024).

Prior research has reported an association between education and overweight or obesity, with the association varying among different populations (Hemmingsson et al., 2021; Hermann et al., 2011; Roskam et al., 2010). While some studies suggest that education has a protective effect on average BMI or the likelihood of obesity (Böckerman et al., 2017; Devaux et al., 2011), our study found weight gain in both educational groups. Additionally, changes in the BMI distribution appeared relatively similar across both educational groups, particularly in recent years, suggesting that the protective effect of higher education may be diminishing or less evident in this context.

Our finding of an increase in BMI across both educational categories, with a more pronounced effect in the middle and top quantiles where concerns about overweight and obesity are the greatest, is in line with trends observed in a study conducted in England (Green et al., 2016). It found an increasing linear trend in BMI for different educational groups among men and women, with a consistent rise in the 95th percentile for all groups over time. Our findings also align with a study done in the USA, which found that increases in body mass index are similar across different educational and income groups over time (Ljungvall & Zimmerman, 2012). The increase in BMI across both educational groups, particularly at the higher end of the BMI distribution, suggests that educational level is unlikely to be a major factor in these observed changes and may not explain growing individual inequalities in BMI. Therefore, a plausible explanation could be a societal change affecting all groups, not just those with lower educational attainment.

### 4.1 Methodological considerations

One of the strengths of this study is the large sample, which includes both genders. Additionally, we examined the change in BMI over a whole decade. However, the sample was limited to individuals in Västerbotten region of Sweden, which may affect the generalizability of the findings to entire Sweden. While this current study focuses on regional data from Västerbotten, we recognize the value of comparing these trends with national-level data. As such, we are planning future work that will examine BMI patterns and trends using national data on pregnant women in Sweden. This will allow us to assess whether the inequalities observed in our study are consistent with broader national trends and to explore potential contributing factors in more depth.

A limitation of this study is that height, weight, and educational attainment were self-reported. This may lead to underreporting of BMI, potentially caused by overreported height or conversely, underreported weight (Gorber et al., 2007; Nyholm et al., 2007), however, we found similar results to previous study using measured weight (Chaparro et al., 2015). In addition, reporting bias might also affect the estimation of educational inequalities among women and men, if there are different patterns of reporting bias in groups with different (high or low) educational attainment (Stommel & Schoenborn, 2009).

Furthermore, bias could be higher among women since they reported their weight retrospectively (three months backward), whereas men reported their current weight. Due to data limitations, our study did not include important factors such as income, preventing us from fully exploring BMI distribution changes between socioeconomic groups. In light of this, education was selected as the primary indicator of socioeconomic position due to its strong and consistent association with health outcomes in Sweden, particularly in relation to BMI. It reflects long-term access to knowledge, health literacy, and lifestyle behaviors (Vogt & Gustafsson, 2022), and is a robust predictor of obesity-related inequalities, with widening disparities observed (Bjermo et al., 2015). Moreover, in a welfare state like Sweden, where income differences are mitigated by social support systems, education offers a more stable indicator of socioeconomic position.

Additionally, our dataset only extends up to 2019. More recent data—particularly covering the COVID-19 pandemic and the post-pandemic period—would offer valuable insights into how BMI patterns may have evolved during this time. We plan to extend our analysis in future studies as newer data become available.

### 4.2 Policy implications

Our primary aim was to document and highlight distributional shifts in BMI over time which has not been addressed previously in Sweden. We believe it is a necessary first step toward understanding broader patterns of health inequality. The results of our study are important for several reasons. First, we observed distinct pattern of weight gain, where expectant parents in higher BMI segments experienced greater increase in weight. This trend poses significant risk due to heightened likelihood of chronic diseases associated with increased BMI. Furthermore, the dramatic increase in weight observed during 2018/19 –just before the COVID-19 pandemic –particularly among women, needs further investigation. The pandemic may have exacerbated this trend, as more women of childbearing age entered pregnancy with a high BMI. This can lead to an increased likelihood of complications during pregnancy and childbirth, and their newborns are more likely to become overweight or obese in the future, perpetuating the cycle of obesity.

While we support population-wide interventions to address overweight and obesity, we also strongly emphasize the need for targeted strategies focusing on subgroups experiencing disproportionate weight gain. For instance, the Inter99 study—a high-risk intervention—demonstrated long-term effectiveness in reducing smoking in Denmark (Pisinger et al., 2008). This intervention demonstrated the effectiveness of targeted lifestyle counselling in reducing cardiovascular risk factors. In this model, high-risk individuals received tailored support through individual and group sessions, with follow-up over several years. This model illustrates how structured, risk-based interventions can inform public health strategies aimed at reducing overweight and obesity in vulnerable subgroups.

To address these risks effectively, it is crucial to better understand the characteristics of individuals at the upper end of the BMI distribution. Such insights will inform the design of targeted interventions aimed at reducing weight in these vulnerable segments of the population.

## 5 Conclusion

Our study showed that BMI among men and women, across varying levels of education, has been increasing over time, with a disproportionately higher rise among those in the upper segments of the BMI distribution. Identifying high-risk groups in vulnerable settings will better equip decision-makers to design targeted interventions aimed at reducing overweight and obesity. Further research is needed to understand the reasons behind the rapid BMI increases, especially among women, over a relatively short period.

## CRediT authorship contribution statement

**Fethi Mohammed Yusuf:** Writing – review & editing, Writing – original draft, Formal analysis, Data curation, Conceptualization. **Anni-Maria Pulkki-Brännström:** Writing – review & editing, Supervision. **Per E. Gustafsson:** Writing – review & editing, Supervision. **Anneli Ivarsson:** Writing – review & editing, Resources. **Marie Lindkvist:** Writing – review & editing. **Masoud Vaezghasemi:** Writing – review & editing, Supervision, Methodology, Funding acquisition, Data curation, Conceptualization.

## Ethical statement

The Swedish Ethical Review Authority approved the study (2010-63- 31M). Only those who had given written informed consent were included in this study.

## Funding

This research was funded in whole by Forte [2022- 00493]. For the purpose of Open Access, the author has applied a CC BY public copyright licence to any Author Accepted Manuscript (AAM) version arising from this submission. The funder was not involved in the conduct of the research and in the preparation of the article.

## Declaration of competing interest

The authors declare that they have no known competing financial interests or personal relationships that could have appeared to influence the work reported in this paper.

## Data Availability

The datasets used for this study are not readily available because Region Västerbotten (the regional health authority) originally collected the data as part of the Salut Programme. We accessed data for the present study after approval from both the Region Västerbotten and the Swedish Ethical Review Authority. The datasets used for this study are available upon request from the last author (Masoud Vaezghasemi), and subject to legal restrictions concerning processing of sensitive personal data.
