# Supplementary material for: Unequal distributional change in body mass index among pre-pregnant women and their male partners in northern Sweden: a quantile regression analysis
Source: SSM Popul Health. 2025 Nov 6;32:101877. doi: 10.1016/j.ssmph.2025.101877 (PMC12663026; doi:10.1016/j.ssmph.2025.101877)
Supplement: Multimedia component 1 [file mmc1.docx]

**Unequal distributional change in body mass index among pre-pregnant women and their male partners in northern Sweden: a quantile regression analysis**

**Contents**

[Table S1 2](#_Toc184937205)

[Table S2: 7](#_Toc184937206)

[Table S3 13](#_Toc184937207)

[Table S4: 18](#_Toc184937208)

[Figure S1 23](#_Toc184937209)

[Figure S2 24](#_Toc184937210)

[Figure S3 25](#_Toc184937211)

[Figure S4 26](#_Toc184937212)

[Figure S5 27](#_Toc184937213)

[Figure S6 28](#_Toc184937214)

Table S1**: Quantile regression coefficients for women, stratified by low and high educational attainment, calculated biennially using 2010/11 as the fixed reference year.**

| **Women** | | | | | **High education** | | | | **Low education** | | | |
| --- | --- | --- | --- | --- | --- | --- | --- | --- | --- | --- | --- | --- |
| **Quantile** | **Estimates** | | | | | | | | | | | |
|  | 2012-13 | 2014-15 | 2016-17 | 2018-19 | 2012-13 | 2014-15 | 2016-17 | 2018-19 | 2012-13 | 2014-15 | 2016-17 | 2018-19 |
| 0,01 | 0,235 | 0,084 | 0,134 | 0,153 | 0,278 | 0,220 | 0,182 | 0,201 | -0,006 | -0,400 | 0,117 | -0,086 |
| 0,02 | 0,205 | 0,154 | 0,238 | 0,373 | 0,015 | 0,035 | 0,028 | 0,110 | 0,290 | 0,096 | 0,131 | 0,434 |
| 0,03 | -0,009 | 0,132 | 0,125 | 0,311 | 0,000 | 0,179 | 0,087 | 0,194 | 0,134 | 0,153 | 0,265 | 0,465 |
| 0,04 | 0,029 | 0,193 | 0,210 | 0,252 | 0,059 | 0,167 | 0,135 | 0,183 | -0,010 | 0,044 | 0,230 | 0,402 |
| 0,05 | 0,085 | 0,203 | 0,246 | 0,291 | 0,005 | 0,080 | 0,050 | 0,103 | 0,027 | 0,206 | 0,319 | 0,380 |
| 0,06 | -0,010 | 0,126 | 0,172 | 0,179 | -0,052 | 0,073 | 0,081 | 0,050 | 0,006 | 0,145 | 0,321 | 0,340 |
| 0,07 | -0,024 | 0,104 | 0,176 | 0,195 | -0,047 | 0,072 | 0,080 | 0,058 | -0,051 | 0,103 | 0,174 | 0,394 |
| 0,08 | -0,065 | 0,133 | 0,114 | 0,182 | -0,036 | 0,150 | 0,077 | 0,083 | 0,037 | 0,152 | 0,275 | 0,466 |
| 0,09 | -0,058 | 0,150 | 0,091 | 0,170 | 0,016 | 0,191 | 0,104 | 0,118 | -0,099 | 0,118 | 0,141 | 0,354 |
| 0,1 | -0,058 | 0,143 | 0,100 | 0,169 | 0,022 | 0,213 | 0,112 | 0,153 | -0,102 | 0,108 | 0,138 | 0,327 |
| 0,11 | -0,066 | 0,140 | 0,100 | 0,166 | -0,019 | 0,161 | 0,093 | 0,123 | -0,172 | -0,002 | 0,057 | 0,254 |
| 0,12 | -0,047 | 0,126 | 0,081 | 0,174 | -0,003 | 0,147 | 0,090 | 0,147 | -0,202 | 0,068 | 0,022 | 0,248 |
| 0,13 | -0,032 | 0,118 | 0,110 | 0,171 | 0,024 | 0,160 | 0,122 | 0,141 | -0,188 | 0,013 | 0,090 | 0,214 |
| 0,14 | -0,018 | 0,103 | 0,120 | 0,207 | 0,041 | 0,161 | 0,147 | 0,161 | -0,163 | -0,004 | 0,059 | 0,297 |
| 0,15 | -0,066 | 0,067 | 0,097 | 0,209 | 0,020 | 0,156 | 0,140 | 0,182 | -0,174 | -0,021 | 0,065 | 0,285 |
| 0,16 | -0,034 | 0,086 | 0,123 | 0,240 | 0,090 | 0,134 | 0,148 | 0,188 | -0,171 | -0,023 | 0,085 | 0,306 |
| 0,17 | 0,007 | 0,068 | 0,108 | 0,235 | 0,092 | 0,130 | 0,120 | 0,216 | -0,167 | -0,024 | 0,144 | 0,270 |
| 0,18 | 0,012 | 0,108 | 0,137 | 0,233 | 0,078 | 0,176 | 0,135 | 0,233 | -0,146 | -0,067 | 0,132 | 0,276 |
| 0,19 | 0,026 | 0,123 | 0,150 | 0,238 | 0,118 | 0,214 | 0,178 | 0,240 | -0,137 | -0,064 | 0,140 | 0,268 |
| 0,2 | 0,039 | 0,135 | 0,182 | 0,237 | 0,088 | 0,200 | 0,165 | 0,229 | -0,032 | -0,020 | 0,150 | 0,360 |
| 0,21 | 0,072 | 0,121 | 0,156 | 0,248 | 0,124 | 0,209 | 0,196 | 0,255 | -0,017 | -0,048 | 0,135 | 0,341 |
| 0,22 | 0,051 | 0,111 | 0,166 | 0,280 | 0,115 | 0,219 | 0,175 | 0,251 | -0,027 | -0,075 | 0,146 | 0,365 |
| 0,23 | 0,030 | 0,109 | 0,168 | 0,253 | 0,088 | 0,219 | 0,183 | 0,275 | -0,045 | -0,097 | 0,115 | 0,337 |
| 0,24 | 0,038 | 0,113 | 0,177 | 0,256 | 0,077 | 0,210 | 0,207 | 0,252 | -0,029 | -0,055 | 0,167 | 0,396 |
| 0,25 | 0,052 | 0,117 | 0,163 | 0,279 | 0,093 | 0,217 | 0,200 | 0,248 | 0,005 | -0,041 | 0,225 | 0,417 |
| 0,26 | 0,050 | 0,123 | 0,199 | 0,321 | 0,092 | 0,185 | 0,183 | 0,259 | -0,026 | 0,029 | 0,171 | 0,402 |
| 0,27 | 0,030 | 0,134 | 0,188 | 0,307 | 0,074 | 0,203 | 0,219 | 0,271 | -0,010 | 0,040 | 0,204 | 0,430 |
| 0,28 | 0,035 | 0,148 | 0,183 | 0,309 | 0,074 | 0,214 | 0,207 | 0,312 | -0,031 | 0,054 | 0,241 | 0,432 |
| 0,29 | 0,041 | 0,124 | 0,157 | 0,301 | 0,078 | 0,211 | 0,207 | 0,296 | -0,059 | 0,070 | 0,217 | 0,455 |
| 0,3 | 0,018 | 0,119 | 0,152 | 0,273 | 0,046 | 0,162 | 0,153 | 0,281 | -0,074 | 0,080 | 0,244 | 0,460 |
| 0,31 | 0,025 | 0,160 | 0,168 | 0,307 | 0,053 | 0,160 | 0,143 | 0,262 | -0,066 | 0,064 | 0,261 | 0,447 |
| 0,32 | 0,030 | 0,154 | 0,166 | 0,298 | 0,047 | 0,168 | 0,149 | 0,237 | -0,083 | 0,099 | 0,302 | 0,467 |
| 0,33 | 0,005 | 0,149 | 0,158 | 0,283 | 0,092 | 0,192 | 0,154 | 0,274 | -0,076 | 0,197 | 0,337 | 0,491 |
| 0,34 | -0,003 | 0,154 | 0,168 | 0,324 | 0,084 | 0,237 | 0,163 | 0,269 | -0,088 | 0,215 | 0,352 | 0,483 |
| 0,35 | -0,016 | 0,167 | 0,160 | 0,323 | 0,022 | 0,191 | 0,131 | 0,226 | -0,116 | 0,170 | 0,304 | 0,454 |
| 0,36 | -0,010 | 0,208 | 0,184 | 0,360 | 0,051 | 0,226 | 0,138 | 0,281 | -0,117 | 0,158 | 0,280 | 0,505 |
| 0,37 | 0,000 | 0,209 | 0,177 | 0,347 | 0,046 | 0,225 | 0,141 | 0,310 | -0,105 | 0,164 | 0,338 | 0,535 |
| 0,38 | -0,023 | 0,182 | 0,178 | 0,337 | 0,047 | 0,239 | 0,150 | 0,320 | -0,104 | 0,158 | 0,354 | 0,519 |
| 0,39 | -0,022 | 0,164 | 0,170 | 0,324 | 0,027 | 0,215 | 0,123 | 0,317 | -0,138 | 0,137 | 0,317 | 0,484 |
| 0,4 | -0,035 | 0,158 | 0,160 | 0,288 | 0,045 | 0,193 | 0,119 | 0,295 | -0,148 | 0,146 | 0,360 | 0,501 |
| 0,41 | -0,034 | 0,175 | 0,164 | 0,304 | 0,013 | 0,190 | 0,121 | 0,293 | -0,162 | 0,137 | 0,354 | 0,512 |
| 0,42 | -0,030 | 0,160 | 0,197 | 0,314 | 0,017 | 0,184 | 0,132 | 0,281 | -0,156 | 0,204 | 0,353 | 0,561 |
| 0,43 | -0,051 | 0,172 | 0,178 | 0,310 | 0,002 | 0,190 | 0,111 | 0,251 | -0,137 | 0,182 | 0,340 | 0,579 |
| 0,44 | -0,054 | 0,145 | 0,163 | 0,309 | 0,023 | 0,175 | 0,124 | 0,246 | -0,111 | 0,136 | 0,300 | 0,569 |
| 0,45 | -0,054 | 0,178 | 0,193 | 0,361 | 0,025 | 0,206 | 0,144 | 0,254 | -0,096 | 0,133 | 0,297 | 0,594 |
| 0,46 | -0,011 | 0,220 | 0,194 | 0,363 | 0,017 | 0,181 | 0,138 | 0,253 | -0,150 | 0,070 | 0,273 | 0,553 |
| 0,47 | 0,000 | 0,232 | 0,199 | 0,409 | 0,027 | 0,182 | 0,122 | 0,258 | -0,184 | 0,124 | 0,272 | 0,542 |
| 0,48 | 0,010 | 0,242 | 0,202 | 0,426 | 0,032 | 0,232 | 0,130 | 0,325 | -0,138 | 0,107 | 0,321 | 0,550 |
| 0,49 | 0,015 | 0,278 | 0,205 | 0,452 | 0,036 | 0,248 | 0,139 | 0,324 | -0,143 | 0,109 | 0,333 | 0,631 |
| 0,5 | 0,014 | 0,275 | 0,182 | 0,474 | 0,069 | 0,297 | 0,158 | 0,404 | -0,192 | 0,073 | 0,280 | 0,606 |
| 0,51 | -0,011 | 0,268 | 0,165 | 0,461 | 0,067 | 0,346 | 0,166 | 0,390 | -0,153 | 0,079 | 0,339 | 0,622 |
| 0,52 | -0,009 | 0,239 | 0,194 | 0,463 | 0,100 | 0,372 | 0,172 | 0,426 | -0,133 | 0,090 | 0,330 | 0,615 |
| 0,53 | -0,055 | 0,205 | 0,152 | 0,451 | 0,112 | 0,395 | 0,187 | 0,482 | -0,130 | 0,056 | 0,298 | 0,578 |
| 0,54 | -0,050 | 0,211 | 0,187 | 0,453 | 0,074 | 0,356 | 0,131 | 0,460 | -0,141 | 0,025 | 0,277 | 0,564 |
| 0,55 | -0,065 | 0,190 | 0,160 | 0,470 | 0,055 | 0,336 | 0,159 | 0,431 | -0,161 | -0,015 | 0,277 | 0,530 |
| 0,56 | -0,040 | 0,180 | 0,193 | 0,525 | 0,062 | 0,317 | 0,149 | 0,438 | -0,216 | -0,060 | 0,258 | 0,516 |
| 0,57 | -0,023 | 0,186 | 0,191 | 0,542 | 0,023 | 0,283 | 0,107 | 0,410 | -0,219 | -0,097 | 0,218 | 0,502 |
| 0,58 | -0,053 | 0,152 | 0,149 | 0,516 | 0,018 | 0,241 | 0,121 | 0,441 | -0,288 | -0,150 | 0,162 | 0,442 |
| 0,59 | -0,050 | 0,174 | 0,132 | 0,495 | 0,039 | 0,262 | 0,131 | 0,495 | -0,223 | -0,138 | 0,112 | 0,432 |
| 0,6 | -0,041 | 0,192 | 0,180 | 0,558 | 0,057 | 0,263 | 0,145 | 0,525 | -0,143 | -0,109 | 0,161 | 0,448 |
| 0,61 | -0,073 | 0,186 | 0,185 | 0,567 | 0,060 | 0,310 | 0,141 | 0,561 | -0,105 | -0,036 | 0,207 | 0,555 |
| 0,62 | -0,091 | 0,181 | 0,155 | 0,567 | 0,014 | 0,296 | 0,103 | 0,523 | -0,017 | -0,040 | 0,172 | 0,596 |
| 0,63 | -0,090 | 0,166 | 0,159 | 0,580 | 0,001 | 0,342 | 0,155 | 0,591 | -0,039 | 0,047 | 0,173 | 0,663 |
| 0,64 | -0,076 | 0,150 | 0,154 | 0,566 | 0,025 | 0,407 | 0,194 | 0,655 | -0,110 | -0,026 | 0,145 | 0,624 |
| 0,65 | -0,067 | 0,156 | 0,148 | 0,544 | 0,010 | 0,376 | 0,170 | 0,650 | -0,113 | -0,061 | 0,200 | 0,669 |
| 0,66 | -0,041 | 0,186 | 0,186 | 0,549 | 0,056 | 0,389 | 0,164 | 0,738 | -0,178 | -0,112 | 0,143 | 0,666 |
| 0,67 | -0,027 | 0,201 | 0,172 | 0,608 | 0,057 | 0,402 | 0,218 | 0,750 | -0,225 | -0,137 | 0,073 | 0,620 |
| 0,68 | 0,027 | 0,221 | 0,187 | 0,683 | 0,046 | 0,367 | 0,253 | 0,706 | -0,167 | -0,130 | 0,047 | 0,704 |
| 0,69 | 0,032 | 0,191 | 0,232 | 0,690 | 0,071 | 0,386 | 0,248 | 0,696 | -0,170 | -0,206 | -0,037 | 0,689 |
| 0,7 | 0,060 | 0,220 | 0,241 | 0,696 | 0,033 | 0,350 | 0,207 | 0,710 | -0,171 | -0,186 | -0,097 | 0,682 |
| 0,71 | 0,005 | 0,182 | 0,208 | 0,760 | 0,096 | 0,356 | 0,252 | 0,767 | -0,160 | -0,120 | -0,052 | 0,752 |
| 0,72 | -0,013 | 0,158 | 0,214 | 0,760 | 0,088 | 0,308 | 0,286 | 0,768 | -0,142 | -0,215 | -0,119 | 0,780 |
| 0,73 | -0,023 | 0,111 | 0,170 | 0,753 | 0,164 | 0,387 | 0,317 | 0,817 | -0,151 | -0,296 | -0,178 | 0,704 |
| 0,74 | -0,052 | 0,071 | 0,107 | 0,731 | 0,160 | 0,387 | 0,330 | 0,923 | -0,081 | -0,248 | -0,168 | 0,689 |
| 0,75 | -0,063 | 0,053 | 0,083 | 0,757 | 0,142 | 0,362 | 0,353 | 0,918 | -0,120 | -0,363 | -0,171 | 0,792 |
| 0,76 | -0,070 | 0,088 | 0,064 | 0,743 | 0,114 | 0,330 | 0,331 | 0,924 | -0,104 | -0,321 | -0,139 | 0,784 |
| 0,77 | -0,082 | 0,068 | 0,049 | 0,802 | 0,073 | 0,241 | 0,261 | 0,867 | -0,110 | -0,229 | -0,189 | 0,927 |
| 0,78 | -0,043 | 0,133 | 0,072 | 0,894 | 0,055 | 0,276 | 0,240 | 0,868 | 0,001 | -0,267 | -0,190 | 0,941 |
| 0,79 | -0,050 | 0,098 | 0,063 | 0,880 | 0,029 | 0,290 | 0,213 | 0,887 | 0,033 | -0,253 | -0,208 | 0,931 |
| 0,8 | -0,013 | 0,109 | 0,068 | 0,947 | 0,012 | 0,305 | 0,195 | 0,917 | 0,157 | -0,141 | -0,089 | 0,914 |
| 0,81 | -0,048 | 0,136 | 0,050 | 0,909 | 0,033 | 0,380 | 0,212 | 1,023 | 0,098 | -0,081 | 0,001 | 1,037 |
| 0,82 | -0,077 | 0,079 | 0,031 | 0,951 | 0,098 | 0,456 | 0,323 | 1,155 | -0,012 | -0,114 | -0,021 | 0,827 |
| 0,83 | -0,042 | 0,110 | 0,047 | 1,041 | 0,063 | 0,503 | 0,331 | 1,230 | 0,117 | -0,107 | -0,102 | 0,901 |
| 0,84 | -0,094 | 0,129 | 0,012 | 0,954 | 0,084 | 0,540 | 0,323 | 1,187 | 0,087 | -0,132 | -0,002 | 1,018 |
| 0,85 | -0,009 | 0,200 | 0,125 | 0,983 | 0,061 | 0,473 | 0,361 | 1,297 | 0,038 | -0,313 | 0,008 | 0,907 |
| 0,86 | 0,021 | 0,175 | 0,054 | 0,947 | -0,002 | 0,558 | 0,318 | 1,260 | -0,115 | -0,287 | -0,135 | 0,614 |
| 0,87 | -0,081 | 0,125 | -0,051 | 0,809 | 0,051 | 0,498 | 0,349 | 1,257 | -0,248 | -0,251 | -0,372 | 0,478 |
| 0,88 | 0,045 | 0,028 | -0,002 | 0,935 | 0,047 | 0,536 | 0,335 | 1,221 | -0,281 | -0,396 | -0,474 | 0,513 |
| 0,89 | 0,062 | 0,054 | 0,047 | 0,910 | 0,015 | 0,570 | 0,255 | 1,102 | -0,310 | -0,274 | -0,456 | 0,549 |
| 0,9 | 0,018 | 0,162 | -0,022 | 0,883 | -0,042 | 0,421 | 0,127 | 1,132 | -0,023 | -0,300 | -0,638 | 0,478 |
| 0,91 | -0,030 | 0,181 | -0,082 | 0,971 | 0,007 | 0,323 | 0,173 | 1,017 | -0,285 | -0,395 | -0,591 | 0,407 |
| 0,92 | -0,101 | 0,031 | -0,301 | 0,922 | 0,246 | 0,569 | 0,316 | 1,291 | -0,432 | -0,281 | -0,913 | 0,351 |
| 0,93 | -0,084 | 0,006 | -0,198 | 0,922 | 0,330 | 0,724 | 0,359 | 1,467 | -0,259 | -0,172 | -0,505 | 0,793 |
| 0,94 | -0,241 | 0,183 | -0,401 | 0,928 | 0,258 | 0,585 | 0,375 | 1,572 | -0,238 | -0,115 | -0,729 | 0,523 |
| 0,95 | -0,125 | 0,204 | -0,240 | 1,075 | 0,010 | 0,606 | 0,226 | 1,588 | -0,357 | -0,271 | -0,268 | 0,481 |
| 0,96 | -0,190 | 0,034 | -0,123 | 0,767 | -0,034 | 0,724 | 0,287 | 1,598 | 0,060 | -0,301 | 0,113 | 0,466 |
| 0,97 | -0,046 | -0,116 | 0,024 | 0,559 | -0,019 | 0,348 | 0,517 | 1,162 | 0,028 | -0,198 | 0,114 | 0,749 |
| 0,98 | -0,120 | -0,148 | -0,007 | 1,005 | -0,006 | 0,111 | 0,090 | 1,374 | 0,183 | -0,570 | 1,498 | 1,194 |
| 0,99 | 1,309 | 0,003 | 1,151 | 1,773 | -0,292 | -0,589 | 0,199 | 1,096 | 1,300 | -0,104 | 1,380 | 2,454 |

Table S2: **Quantile regression coefficients for women, stratified by low and high educational attainment, calculated biennially using variable reference time periods (period-to-period analysis).**

| **Women** | | | | | **High education** | | | | **Low education** | | | |
| --- | --- | --- | --- | --- | --- | --- | --- | --- | --- | --- | --- | --- |
| **Quantile** | **Estimate** | | | | | | | | | | | |
|  | 2010-13 | 2012-15 | 2014-17 | 2016-19 | 2010-13 | 2012-15 | 2014-17 | 2016-19 | 2010-13 | 2012-15 | 2014-17 | 2016-19 |
| 0,01 | 0,235 | -0,151 | 0,050 | 0,019 | 0,278 | -0,058 | -0,038 | 0,019 | -0,006 | -0,394 | 0,516 | -0,202 |
| 0,02 | 0,205 | -0,051 | 0,084 | 0,135 | 0,015 | 0,020 | -0,008 | 0,083 | 0,290 | -0,194 | 0,035 | 0,302 |
| 0,03 | -0,009 | 0,141 | -0,007 | 0,186 | 0,000 | 0,179 | -0,092 | 0,107 | 0,134 | 0,019 | 0,112 | 0,200 |
| 0,04 | 0,029 | 0,164 | 0,017 | 0,042 | 0,059 | 0,107 | -0,032 | 0,048 | -0,010 | 0,054 | 0,186 | 0,172 |
| 0,05 | 0,085 | 0,118 | 0,043 | 0,045 | 0,005 | 0,076 | -0,031 | 0,054 | 0,027 | 0,179 | 0,112 | 0,061 |
| 0,06 | -0,010 | 0,136 | 0,046 | 0,007 | -0,052 | 0,125 | 0,008 | -0,031 | 0,006 | 0,139 | 0,176 | 0,019 |
| 0,07 | -0,024 | 0,128 | 0,072 | 0,019 | -0,047 | 0,119 | 0,008 | -0,022 | -0,051 | 0,153 | 0,071 | 0,221 |
| 0,08 | -0,065 | 0,198 | -0,018 | 0,068 | -0,036 | 0,186 | -0,073 | 0,005 | 0,037 | 0,115 | 0,123 | 0,191 |
| 0,09 | -0,058 | 0,208 | -0,059 | 0,079 | 0,016 | 0,175 | -0,086 | 0,014 | -0,099 | 0,217 | 0,023 | 0,213 |
| 0,1 | -0,058 | 0,202 | -0,043 | 0,069 | 0,022 | 0,190 | -0,100 | 0,041 | -0,102 | 0,210 | 0,030 | 0,188 |
| 0,11 | -0,066 | 0,206 | -0,040 | 0,067 | -0,019 | 0,181 | -0,068 | 0,030 | -0,172 | 0,170 | 0,058 | 0,197 |
| 0,12 | -0,047 | 0,172 | -0,045 | 0,094 | -0,003 | 0,151 | -0,057 | 0,057 | -0,202 | 0,269 | -0,046 | 0,226 |
| 0,13 | -0,032 | 0,150 | -0,008 | 0,060 | 0,024 | 0,136 | -0,037 | 0,019 | -0,188 | 0,201 | 0,077 | 0,123 |
| 0,14 | -0,018 | 0,122 | 0,017 | 0,087 | 0,041 | 0,121 | -0,014 | 0,014 | -0,163 | 0,160 | 0,063 | 0,238 |
| 0,15 | -0,066 | 0,133 | 0,030 | 0,112 | 0,020 | 0,136 | -0,016 | 0,042 | -0,174 | 0,153 | 0,086 | 0,220 |
| 0,16 | -0,034 | 0,120 | 0,037 | 0,117 | 0,090 | 0,044 | 0,014 | 0,040 | -0,171 | 0,147 | 0,109 | 0,220 |
| 0,17 | 0,007 | 0,060 | 0,040 | 0,127 | 0,092 | 0,039 | -0,010 | 0,096 | -0,167 | 0,143 | 0,168 | 0,126 |
| 0,18 | 0,012 | 0,095 | 0,029 | 0,097 | 0,078 | 0,097 | -0,041 | 0,098 | -0,146 | 0,079 | 0,199 | 0,144 |
| 0,19 | 0,026 | 0,097 | 0,027 | 0,088 | 0,118 | 0,096 | -0,036 | 0,062 | -0,137 | 0,073 | 0,204 | 0,128 |
| 0,2 | 0,039 | 0,095 | 0,048 | 0,055 | 0,088 | 0,113 | -0,033 | 0,061 | -0,032 | 0,013 | 0,170 | 0,210 |
| 0,21 | 0,072 | 0,049 | 0,035 | 0,093 | 0,124 | 0,085 | -0,012 | 0,059 | -0,017 | -0,031 | 0,182 | 0,207 |
| 0,22 | 0,051 | 0,060 | 0,056 | 0,114 | 0,115 | 0,104 | -0,044 | 0,076 | -0,027 | -0,049 | 0,222 | 0,218 |
| 0,23 | 0,030 | 0,079 | 0,059 | 0,085 | 0,088 | 0,131 | -0,037 | 0,092 | -0,045 | -0,052 | 0,211 | 0,222 |
| 0,24 | 0,038 | 0,075 | 0,064 | 0,078 | 0,077 | 0,134 | -0,004 | 0,046 | -0,029 | -0,026 | 0,222 | 0,229 |
| 0,25 | 0,052 | 0,065 | 0,046 | 0,116 | 0,093 | 0,124 | -0,017 | 0,049 | 0,005 | -0,046 | 0,265 | 0,192 |
| 0,26 | 0,050 | 0,074 | 0,076 | 0,121 | 0,092 | 0,092 | -0,001 | 0,076 | -0,026 | 0,055 | 0,142 | 0,231 |
| 0,27 | 0,030 | 0,104 | 0,053 | 0,120 | 0,074 | 0,128 | 0,016 | 0,052 | -0,010 | 0,050 | 0,164 | 0,226 |
| 0,28 | 0,035 | 0,113 | 0,034 | 0,127 | 0,074 | 0,140 | -0,007 | 0,105 | -0,031 | 0,085 | 0,188 | 0,190 |
| 0,29 | 0,041 | 0,083 | 0,033 | 0,144 | 0,078 | 0,133 | -0,004 | 0,089 | -0,059 | 0,129 | 0,147 | 0,239 |
| 0,3 | 0,018 | 0,101 | 0,034 | 0,120 | 0,046 | 0,116 | -0,009 | 0,128 | -0,074 | 0,154 | 0,164 | 0,216 |
| 0,31 | 0,025 | 0,135 | 0,008 | 0,140 | 0,053 | 0,107 | -0,017 | 0,119 | -0,066 | 0,129 | 0,197 | 0,186 |
| 0,32 | 0,030 | 0,124 | 0,012 | 0,133 | 0,047 | 0,121 | -0,018 | 0,088 | -0,083 | 0,182 | 0,203 | 0,165 |
| 0,33 | 0,005 | 0,144 | 0,009 | 0,124 | 0,092 | 0,100 | -0,038 | 0,120 | -0,076 | 0,273 | 0,140 | 0,154 |
| 0,34 | -0,003 | 0,157 | 0,014 | 0,156 | 0,084 | 0,153 | -0,074 | 0,107 | -0,088 | 0,303 | 0,137 | 0,131 |
| 0,35 | -0,016 | 0,183 | -0,007 | 0,163 | 0,022 | 0,169 | -0,059 | 0,095 | -0,116 | 0,285 | 0,134 | 0,150 |
| 0,36 | -0,010 | 0,218 | -0,024 | 0,176 | 0,051 | 0,176 | -0,089 | 0,143 | -0,117 | 0,274 | 0,122 | 0,225 |
| 0,37 | 0,000 | 0,209 | -0,032 | 0,170 | 0,046 | 0,179 | -0,084 | 0,170 | -0,105 | 0,269 | 0,174 | 0,197 |
| 0,38 | -0,023 | 0,204 | -0,003 | 0,159 | 0,047 | 0,192 | -0,089 | 0,170 | -0,104 | 0,262 | 0,195 | 0,165 |
| 0,39 | -0,022 | 0,186 | 0,006 | 0,154 | 0,027 | 0,188 | -0,092 | 0,194 | -0,138 | 0,275 | 0,180 | 0,167 |
| 0,4 | -0,035 | 0,193 | 0,002 | 0,129 | 0,045 | 0,148 | -0,074 | 0,176 | -0,148 | 0,294 | 0,214 | 0,141 |
| 0,41 | -0,034 | 0,209 | -0,011 | 0,139 | 0,013 | 0,177 | -0,069 | 0,173 | -0,162 | 0,299 | 0,216 | 0,159 |
| 0,42 | -0,030 | 0,190 | 0,036 | 0,117 | 0,017 | 0,167 | -0,052 | 0,149 | -0,156 | 0,360 | 0,149 | 0,208 |
| 0,43 | -0,051 | 0,223 | 0,006 | 0,132 | 0,002 | 0,187 | -0,079 | 0,140 | -0,137 | 0,319 | 0,158 | 0,239 |
| 0,44 | -0,054 | 0,199 | 0,018 | 0,146 | 0,023 | 0,152 | -0,052 | 0,123 | -0,111 | 0,247 | 0,165 | 0,269 |
| 0,45 | -0,054 | 0,231 | 0,016 | 0,168 | 0,025 | 0,182 | -0,062 | 0,109 | -0,096 | 0,230 | 0,163 | 0,297 |
| 0,46 | -0,011 | 0,232 | -0,027 | 0,169 | 0,017 | 0,164 | -0,043 | 0,115 | -0,150 | 0,220 | 0,203 | 0,280 |
| 0,47 | 0,000 | 0,232 | -0,033 | 0,210 | 0,027 | 0,155 | -0,060 | 0,136 | -0,184 | 0,308 | 0,148 | 0,270 |
| 0,48 | 0,010 | 0,232 | -0,039 | 0,223 | 0,032 | 0,199 | -0,101 | 0,194 | -0,138 | 0,245 | 0,213 | 0,229 |
| 0,49 | 0,015 | 0,263 | -0,073 | 0,247 | 0,036 | 0,212 | -0,109 | 0,185 | -0,143 | 0,252 | 0,223 | 0,299 |
| 0,5 | 0,014 | 0,262 | -0,093 | 0,291 | 0,069 | 0,228 | -0,140 | 0,246 | -0,192 | 0,266 | 0,207 | 0,318 |
| 0,51 | -0,011 | 0,279 | -0,103 | 0,297 | 0,067 | 0,278 | -0,180 | 0,224 | -0,153 | 0,233 | 0,260 | 0,283 |
| 0,52 | -0,009 | 0,249 | -0,045 | 0,269 | 0,100 | 0,272 | -0,200 | 0,254 | -0,133 | 0,223 | 0,241 | 0,285 |
| 0,53 | -0,055 | 0,261 | -0,053 | 0,299 | 0,112 | 0,283 | -0,209 | 0,295 | -0,130 | 0,186 | 0,241 | 0,280 |
| 0,54 | -0,050 | 0,262 | -0,024 | 0,265 | 0,074 | 0,282 | -0,225 | 0,329 | -0,141 | 0,166 | 0,253 | 0,287 |
| 0,55 | -0,065 | 0,255 | -0,030 | 0,310 | 0,055 | 0,281 | -0,177 | 0,272 | -0,161 | 0,146 | 0,292 | 0,253 |
| 0,56 | -0,040 | 0,221 | 0,013 | 0,332 | 0,062 | 0,256 | -0,168 | 0,289 | -0,216 | 0,156 | 0,318 | 0,258 |
| 0,57 | -0,023 | 0,209 | 0,005 | 0,350 | 0,023 | 0,259 | -0,176 | 0,303 | -0,219 | 0,122 | 0,316 | 0,284 |
| 0,58 | -0,053 | 0,205 | -0,003 | 0,367 | 0,018 | 0,223 | -0,120 | 0,320 | -0,288 | 0,137 | 0,312 | 0,280 |
| 0,59 | -0,050 | 0,223 | -0,042 | 0,363 | 0,039 | 0,223 | -0,131 | 0,364 | -0,223 | 0,085 | 0,250 | 0,320 |
| 0,6 | -0,041 | 0,234 | -0,012 | 0,377 | 0,057 | 0,206 | -0,118 | 0,380 | -0,143 | 0,034 | 0,270 | 0,287 |
| 0,61 | -0,073 | 0,258 | -0,001 | 0,383 | 0,060 | 0,250 | -0,169 | 0,420 | -0,105 | 0,069 | 0,243 | 0,348 |
| 0,62 | -0,091 | 0,271 | -0,025 | 0,412 | 0,014 | 0,283 | -0,193 | 0,420 | -0,017 | -0,023 | 0,212 | 0,424 |
| 0,63 | -0,090 | 0,256 | -0,007 | 0,421 | 0,001 | 0,342 | -0,187 | 0,435 | -0,039 | 0,086 | 0,126 | 0,490 |
| 0,64 | -0,076 | 0,226 | 0,004 | 0,412 | 0,025 | 0,382 | -0,213 | 0,461 | -0,110 | 0,084 | 0,171 | 0,479 |
| 0,65 | -0,067 | 0,223 | -0,008 | 0,396 | 0,010 | 0,367 | -0,206 | 0,479 | -0,113 | 0,052 | 0,260 | 0,470 |
| 0,66 | -0,041 | 0,227 | 0,000 | 0,363 | 0,056 | 0,333 | -0,225 | 0,575 | -0,178 | 0,067 | 0,255 | 0,523 |
| 0,67 | -0,027 | 0,228 | -0,028 | 0,435 | 0,057 | 0,345 | -0,185 | 0,533 | -0,225 | 0,088 | 0,210 | 0,547 |
| 0,68 | 0,027 | 0,194 | -0,034 | 0,495 | 0,046 | 0,322 | -0,114 | 0,453 | -0,167 | 0,036 | 0,178 | 0,657 |
| 0,69 | 0,032 | 0,159 | 0,041 | 0,458 | 0,071 | 0,314 | -0,138 | 0,448 | -0,170 | -0,036 | 0,169 | 0,726 |
| 0,7 | 0,060 | 0,160 | 0,021 | 0,455 | 0,033 | 0,318 | -0,143 | 0,502 | -0,171 | -0,007 | 0,089 | 0,779 |
| 0,71 | 0,005 | 0,177 | 0,026 | 0,552 | 0,096 | 0,260 | -0,104 | 0,514 | -0,160 | 0,040 | 0,068 | 0,803 |
| 0,72 | -0,013 | 0,171 | 0,055 | 0,547 | 0,088 | 0,220 | -0,021 | 0,481 | -0,142 | -0,072 | 0,095 | 0,899 |
| 0,73 | -0,023 | 0,135 | 0,059 | 0,583 | 0,164 | 0,224 | -0,070 | 0,499 | -0,151 | -0,145 | 0,118 | 0,882 |
| 0,74 | -0,052 | 0,122 | 0,037 | 0,623 | 0,160 | 0,227 | -0,057 | 0,593 | -0,081 | -0,167 | 0,080 | 0,857 |
| 0,75 | -0,063 | 0,116 | 0,030 | 0,674 | 0,142 | 0,220 | -0,009 | 0,565 | -0,120 | -0,244 | 0,192 | 0,963 |
| 0,76 | -0,070 | 0,158 | -0,024 | 0,679 | 0,114 | 0,216 | 0,001 | 0,593 | -0,104 | -0,218 | 0,182 | 0,923 |
| 0,77 | -0,082 | 0,151 | -0,019 | 0,752 | 0,073 | 0,167 | 0,021 | 0,606 | -0,110 | -0,119 | 0,040 | 1,117 |
| 0,78 | -0,043 | 0,177 | -0,061 | 0,821 | 0,055 | 0,221 | -0,037 | 0,629 | 0,001 | -0,268 | 0,077 | 1,132 |
| 0,79 | -0,050 | 0,148 | -0,034 | 0,817 | 0,029 | 0,261 | -0,077 | 0,675 | 0,033 | -0,286 | 0,045 | 1,139 |
| 0,8 | -0,013 | 0,122 | -0,041 | 0,879 | 0,012 | 0,294 | -0,105 | 0,717 | 0,157 | -0,298 | 0,052 | 1,004 |
| 0,81 | -0,048 | 0,184 | -0,085 | 0,858 | 0,033 | 0,347 | -0,168 | 0,811 | 0,098 | -0,179 | 0,081 | 1,037 |
| 0,82 | -0,077 | 0,156 | -0,049 | 0,920 | 0,098 | 0,358 | -0,133 | 0,832 | -0,012 | -0,103 | 0,094 | 0,848 |
| 0,83 | -0,042 | 0,152 | -0,063 | 0,994 | 0,063 | 0,440 | -0,172 | 0,899 | 0,117 | -0,224 | 0,005 | 1,003 |
| 0,84 | -0,094 | 0,223 | -0,117 | 0,942 | 0,084 | 0,456 | -0,217 | 0,864 | 0,087 | -0,219 | 0,130 | 1,020 |
| 0,85 | -0,009 | 0,209 | -0,075 | 0,858 | 0,061 | 0,412 | -0,112 | 0,936 | 0,038 | -0,351 | 0,321 | 0,899 |
| 0,86 | 0,021 | 0,155 | -0,121 | 0,892 | -0,002 | 0,560 | -0,239 | 0,942 | -0,115 | -0,172 | 0,152 | 0,748 |
| 0,87 | -0,081 | 0,206 | -0,176 | 0,860 | 0,051 | 0,447 | -0,149 | 0,907 | -0,248 | -0,003 | -0,121 | 0,850 |
| 0,88 | 0,045 | -0,017 | -0,030 | 0,937 | 0,047 | 0,489 | -0,200 | 0,885 | -0,281 | -0,115 | -0,077 | 0,986 |
| 0,89 | 0,062 | -0,007 | -0,007 | 0,863 | 0,015 | 0,555 | -0,315 | 0,847 | -0,310 | 0,036 | -0,182 | 1,005 |
| 0,9 | 0,018 | 0,144 | -0,184 | 0,905 | -0,042 | 0,463 | -0,295 | 1,005 | -0,023 | -0,242 | -0,338 | 1,116 |
| 0,91 | -0,030 | 0,211 | -0,263 | 1,053 | 0,007 | 0,317 | -0,150 | 0,844 | -0,285 | -0,109 | -0,196 | 0,997 |
| 0,92 | -0,101 | 0,132 | -0,331 | 1,223 | 0,246 | 0,323 | -0,253 | 0,975 | -0,432 | 0,151 | -0,632 | 1,264 |
| 0,93 | -0,084 | 0,090 | -0,203 | 1,119 | 0,330 | 0,394 | -0,365 | 1,108 | -0,259 | 0,087 | -0,332 | 1,297 |
| 0,94 | -0,241 | 0,424 | -0,583 | 1,329 | 0,258 | 0,327 | -0,210 | 1,197 | -0,238 | 0,123 | -0,614 | 1,251 |
| 0,95 | -0,125 | 0,328 | -0,444 | 1,315 | 0,010 | 0,595 | -0,380 | 1,362 | -0,357 | 0,087 | 0,003 | 0,748 |
| 0,96 | -0,190 | 0,224 | -0,157 | 0,890 | -0,034 | 0,758 | -0,437 | 1,311 | 0,060 | -0,361 | 0,415 | 0,352 |
| 0,97 | -0,046 | -0,071 | 0,140 | 0,535 | -0,019 | 0,367 | 0,168 | 0,645 | 0,028 | -0,227 | 0,312 | 0,635 |
| 0,98 | -0,120 | -0,028 | 0,142 | 1,012 | -0,006 | 0,117 | -0,021 | 1,284 | 0,183 | -0,753 | 2,067 | -0,303 |
| 0,99 | 1,309 | -1,306 | 1,148 | 0,622 | -0,292 | -0,297 | 0,788 | 0,896 | 1,300 | -1,403 | 1,484 | 1,074 |

Table S3: **Quantile regression coefficients for men, stratified by low and high educational attainment, calculated biennially using 2010/11 as the fixed reference year.**

| **Men** | | | | | **High education** | | | | **Low education** | | | |
| --- | --- | --- | --- | --- | --- | --- | --- | --- | --- | --- | --- | --- |
| **Quantile** | **Estimate** | | | | | | | | | | | |
|  | 2012-13 | 2014-15 | 2016-17 | 2018-19 | 2012-13 | 2014-15 | 2016-17 | 2018-19 | 2012-13 | 2014-15 | 2016-17 | 2018-19 |
| 0,01 | 0,037 | -0,518 | -0,054 | -0,441 | -0,155 | -0,855 | 0,174 | -0,578 | 0,166 | -0,056 | -0,536 | -0,240 |
| 0,02 | -0,069 | -0,213 | -0,079 | -0,285 | 0,020 | -0,648 | 0,112 | -0,211 | -0,108 | -0,002 | -0,385 | -0,354 |
| 0,03 | -0,002 | -0,015 | -0,009 | -0,197 | -0,029 | -0,314 | 0,062 | -0,255 | -0,072 | 0,033 | -0,085 | -0,016 |
| 0,04 | -0,116 | -0,124 | -0,054 | -0,243 | 0,019 | -0,132 | 0,110 | -0,296 | -0,206 | -0,200 | -0,180 | -0,114 |
| 0,05 | -0,019 | -0,066 | -0,044 | -0,119 | -0,080 | -0,139 | 0,021 | -0,350 | -0,104 | -0,059 | -0,179 | -0,059 |
| 0,06 | 0,023 | -0,075 | -0,006 | -0,062 | 0,065 | -0,049 | 0,145 | -0,120 | -0,138 | -0,104 | -0,179 | -0,035 |
| 0,07 | 0,026 | -0,038 | 0,009 | -0,055 | 0,105 | -0,087 | 0,105 | -0,065 | -0,026 | 0,037 | -0,129 | 0,008 |
| 0,08 | 0,067 | 0,018 | 0,005 | -0,040 | 0,125 | 0,012 | 0,101 | -0,088 | 0,025 | 0,065 | -0,126 | 0,113 |
| 0,09 | 0,036 | 0,056 | 0,015 | -0,041 | 0,114 | 0,005 | 0,120 | -0,074 | -0,034 | 0,113 | -0,085 | 0,160 |
| 0,10 | 0,020 | 0,051 | 0,020 | -0,022 | 0,074 | 0,019 | 0,126 | -0,089 | 0,000 | 0,087 | -0,037 | 0,159 |
| 0,11 | 0,012 | 0,050 | 0,032 | -0,057 | 0,089 | 0,058 | 0,180 | -0,069 | -0,024 | 0,098 | -0,089 | 0,099 |
| 0,12 | 0,001 | 0,046 | 0,028 | -0,052 | 0,065 | 0,044 | 0,146 | -0,074 | -0,025 | 0,093 | -0,030 | 0,140 |
| 0,13 | 0,010 | 0,034 | 0,042 | -0,069 | 0,051 | 0,031 | 0,135 | -0,146 | -0,101 | 0,034 | -0,043 | 0,171 |
| 0,14 | 0,027 | 0,064 | 0,076 | -0,060 | 0,044 | 0,009 | 0,146 | -0,171 | -0,158 | 0,034 | -0,071 | 0,117 |
| 0,15 | 0,023 | 0,039 | 0,090 | -0,031 | 0,068 | -0,011 | 0,138 | -0,218 | -0,204 | 0,042 | -0,054 | 0,084 |
| 0,16 | -0,034 | -0,002 | 0,067 | -0,057 | 0,106 | 0,042 | 0,122 | -0,196 | -0,206 | 0,016 | -0,055 | 0,096 |
| 0,17 | -0,045 | 0,043 | 0,074 | -0,001 | 0,087 | 0,018 | 0,164 | -0,191 | -0,124 | 0,073 | 0,022 | 0,114 |
| 0,18 | -0,054 | 0,037 | 0,069 | -0,013 | 0,069 | -0,007 | 0,170 | -0,197 | -0,134 | 0,035 | 0,054 | 0,058 |
| 0,19 | -0,062 | -0,003 | 0,069 | -0,049 | 0,080 | 0,012 | 0,185 | -0,169 | -0,105 | 0,098 | 0,051 | 0,054 |
| 0,20 | 0,000 | 0,027 | 0,136 | -0,021 | 0,082 | 0,043 | 0,198 | -0,115 | -0,096 | 0,088 | 0,064 | 0,044 |
| 0,21 | -0,006 | 0,033 | 0,139 | 0,001 | 0,056 | 0,035 | 0,160 | -0,106 | -0,099 | 0,093 | 0,083 | 0,074 |
| 0,22 | -0,015 | 0,085 | 0,122 | 0,014 | 0,020 | -0,008 | 0,154 | -0,130 | -0,065 | 0,099 | 0,093 | 0,081 |
| 0,23 | -0,011 | 0,082 | 0,118 | -0,008 | 0,054 | -0,006 | 0,205 | -0,106 | -0,060 | 0,091 | 0,070 | 0,146 |
| 0,24 | 0,051 | 0,097 | 0,130 | 0,000 | 0,047 | 0,009 | 0,204 | -0,096 | -0,031 | 0,105 | 0,109 | 0,164 |
| 0,25 | 0,026 | 0,105 | 0,119 | 0,001 | 0,069 | 0,033 | 0,188 | -0,091 | -0,040 | 0,091 | 0,101 | 0,149 |
| 0,26 | 0,030 | 0,093 | 0,104 | 0,020 | 0,116 | 0,122 | 0,198 | -0,015 | -0,036 | 0,119 | 0,095 | 0,191 |
| 0,27 | 0,022 | 0,061 | 0,072 | 0,025 | 0,144 | 0,098 | 0,188 | -0,018 | -0,020 | 0,085 | 0,090 | 0,205 |
| 0,28 | 0,032 | 0,075 | 0,094 | 0,064 | 0,134 | 0,126 | 0,191 | -0,011 | 0,006 | 0,083 | 0,070 | 0,205 |
| 0,29 | 0,032 | 0,071 | 0,098 | 0,069 | 0,151 | 0,155 | 0,194 | 0,007 | 0,013 | 0,073 | 0,079 | 0,195 |
| 0,30 | 0,030 | 0,050 | 0,088 | 0,054 | 0,136 | 0,115 | 0,135 | -0,015 | 0,026 | 0,089 | 0,092 | 0,254 |
| 0,31 | 0,038 | 0,049 | 0,080 | 0,080 | 0,099 | 0,074 | 0,099 | -0,009 | 0,015 | 0,071 | 0,064 | 0,273 |
| 0,32 | 0,051 | 0,032 | 0,082 | 0,106 | 0,105 | 0,050 | 0,104 | 0,004 | -0,005 | 0,064 | 0,078 | 0,258 |
| 0,33 | 0,041 | 0,019 | 0,058 | 0,110 | 0,081 | 0,079 | 0,102 | -0,003 | -0,006 | 0,033 | 0,121 | 0,311 |
| 0,34 | 0,038 | 0,032 | 0,068 | 0,119 | 0,051 | 0,032 | 0,072 | -0,016 | 0,012 | 0,066 | 0,123 | 0,288 |
| 0,35 | 0,025 | 0,018 | 0,044 | 0,139 | 0,069 | -0,004 | 0,075 | -0,009 | 0,008 | 0,076 | 0,140 | 0,314 |
| 0,36 | 0,026 | 0,014 | 0,050 | 0,139 | 0,072 | -0,003 | 0,085 | -0,009 | 0,016 | 0,054 | 0,115 | 0,294 |
| 0,37 | 0,019 | 0,022 | 0,062 | 0,150 | 0,094 | -0,019 | 0,100 | 0,035 | 0,073 | 0,074 | 0,106 | 0,276 |
| 0,38 | 0,042 | 0,035 | 0,106 | 0,161 | 0,044 | -0,041 | 0,055 | 0,026 | 0,060 | 0,082 | 0,126 | 0,280 |
| 0,39 | 0,040 | 0,026 | 0,100 | 0,180 | 0,031 | -0,045 | 0,050 | 0,041 | 0,062 | 0,066 | 0,151 | 0,268 |
| 0,40 | 0,030 | 0,020 | 0,091 | 0,165 | 0,040 | -0,013 | 0,044 | 0,074 | 0,086 | 0,096 | 0,185 | 0,294 |
| 0,41 | 0,052 | 0,022 | 0,087 | 0,195 | 0,057 | -0,036 | 0,049 | 0,074 | 0,093 | 0,090 | 0,173 | 0,326 |
| 0,42 | 0,062 | 0,029 | 0,097 | 0,194 | 0,062 | 0,002 | 0,051 | 0,056 | 0,104 | 0,117 | 0,175 | 0,321 |
| 0,43 | 0,054 | 0,041 | 0,105 | 0,188 | 0,097 | 0,042 | 0,109 | 0,119 | 0,105 | 0,125 | 0,172 | 0,324 |
| 0,44 | 0,039 | 0,033 | 0,108 | 0,179 | 0,111 | 0,041 | 0,100 | 0,125 | 0,106 | 0,169 | 0,168 | 0,341 |
| 0,45 | 0,044 | 0,046 | 0,128 | 0,176 | 0,061 | -0,013 | 0,081 | 0,081 | 0,125 | 0,170 | 0,159 | 0,341 |
| 0,46 | 0,044 | 0,039 | 0,124 | 0,181 | 0,051 | 0,001 | 0,080 | 0,083 | 0,115 | 0,114 | 0,158 | 0,345 |
| 0,47 | 0,052 | 0,058 | 0,124 | 0,201 | 0,058 | -0,001 | 0,077 | 0,095 | 0,133 | 0,153 | 0,209 | 0,355 |
| 0,48 | 0,063 | 0,042 | 0,104 | 0,200 | 0,051 | -0,001 | 0,088 | 0,114 | 0,130 | 0,210 | 0,211 | 0,388 |
| 0,49 | 0,056 | 0,065 | 0,091 | 0,173 | 0,022 | -0,008 | 0,090 | 0,095 | 0,107 | 0,165 | 0,210 | 0,367 |
| 0,50 | 0,068 | 0,054 | 0,084 | 0,189 | -0,001 | -0,023 | 0,069 | 0,084 | 0,147 | 0,174 | 0,280 | 0,388 |
| 0,51 | 0,069 | 0,051 | 0,085 | 0,201 | -0,010 | -0,017 | 0,084 | 0,062 | 0,170 | 0,129 | 0,266 | 0,362 |
| 0,52 | 0,090 | 0,064 | 0,128 | 0,224 | 0,000 | 0,000 | 0,097 | 0,102 | 0,205 | 0,144 | 0,336 | 0,416 |
| 0,53 | 0,073 | 0,091 | 0,125 | 0,221 | -0,004 | -0,004 | 0,066 | 0,090 | 0,186 | 0,130 | 0,369 | 0,383 |
| 0,54 | 0,085 | 0,107 | 0,124 | 0,253 | 0,014 | -0,017 | 0,038 | 0,076 | 0,217 | 0,179 | 0,380 | 0,415 |
| 0,55 | 0,093 | 0,114 | 0,165 | 0,275 | 0,021 | -0,020 | 0,059 | 0,068 | 0,205 | 0,162 | 0,358 | 0,448 |
| 0,56 | 0,125 | 0,092 | 0,174 | 0,294 | 0,029 | -0,022 | 0,051 | 0,092 | 0,205 | 0,136 | 0,347 | 0,486 |
| 0,57 | 0,132 | 0,089 | 0,200 | 0,293 | 0,048 | -0,015 | 0,051 | 0,085 | 0,156 | 0,145 | 0,310 | 0,431 |
| 0,58 | 0,133 | 0,075 | 0,215 | 0,295 | 0,034 | -0,025 | 0,050 | 0,102 | 0,143 | 0,170 | 0,358 | 0,450 |
| 0,59 | 0,119 | 0,065 | 0,233 | 0,291 | 0,054 | 0,012 | 0,075 | 0,115 | 0,114 | 0,146 | 0,315 | 0,399 |
| 0,60 | 0,059 | 0,047 | 0,189 | 0,259 | 0,061 | 0,025 | 0,053 | 0,145 | 0,081 | 0,148 | 0,268 | 0,366 |
| 0,61 | 0,064 | 0,051 | 0,210 | 0,282 | 0,092 | 0,088 | 0,115 | 0,221 | 0,078 | 0,166 | 0,264 | 0,379 |
| 0,62 | 0,065 | 0,082 | 0,198 | 0,320 | 0,086 | 0,067 | 0,100 | 0,245 | 0,044 | 0,138 | 0,232 | 0,334 |
| 0,63 | 0,024 | 0,067 | 0,205 | 0,274 | 0,036 | 0,011 | 0,061 | 0,212 | 0,040 | 0,144 | 0,290 | 0,324 |
| 0,64 | 0,008 | 0,055 | 0,218 | 0,301 | -0,015 | -0,070 | 0,014 | 0,164 | 0,030 | 0,143 | 0,284 | 0,341 |
| 0,65 | 0,028 | 0,102 | 0,203 | 0,290 | -0,030 | -0,059 | 0,020 | 0,150 | 0,029 | 0,122 | 0,256 | 0,423 |
| 0,66 | 0,024 | 0,104 | 0,206 | 0,276 | -0,010 | 0,007 | 0,103 | 0,190 | -0,009 | 0,112 | 0,267 | 0,404 |
| 0,67 | 0,006 | 0,101 | 0,204 | 0,272 | -0,020 | 0,006 | 0,119 | 0,202 | 0,005 | 0,095 | 0,258 | 0,389 |
| 0,68 | -0,018 | 0,099 | 0,214 | 0,246 | -0,032 | 0,029 | 0,120 | 0,199 | -0,005 | 0,090 | 0,252 | 0,453 |
| 0,69 | 0,001 | 0,129 | 0,248 | 0,280 | -0,071 | 0,001 | 0,136 | 0,174 | 0,002 | 0,133 | 0,260 | 0,495 |
| 0,70 | 0,010 | 0,137 | 0,260 | 0,336 | -0,069 | 0,029 | 0,181 | 0,225 | -0,010 | 0,145 | 0,290 | 0,472 |
| 0,71 | -0,030 | 0,081 | 0,235 | 0,309 | -0,010 | 0,051 | 0,185 | 0,249 | -0,030 | 0,141 | 0,281 | 0,444 |
| 0,72 | -0,035 | 0,063 | 0,237 | 0,310 | -0,032 | 0,099 | 0,219 | 0,236 | -0,049 | 0,164 | 0,266 | 0,492 |
| 0,73 | -0,016 | 0,074 | 0,266 | 0,364 | -0,033 | 0,105 | 0,192 | 0,244 | -0,051 | 0,189 | 0,253 | 0,574 |
| 0,74 | -0,032 | 0,078 | 0,251 | 0,354 | -0,005 | 0,131 | 0,239 | 0,289 | -0,001 | 0,216 | 0,280 | 0,619 |
| 0,75 | -0,015 | 0,107 | 0,293 | 0,378 | -0,025 | 0,134 | 0,266 | 0,275 | 0,025 | 0,181 | 0,276 | 0,669 |
| 0,76 | 0,004 | 0,177 | 0,321 | 0,373 | -0,074 | 0,094 | 0,256 | 0,255 | 0,118 | 0,223 | 0,245 | 0,705 |
| 0,77 | 0,025 | 0,206 | 0,332 | 0,432 | -0,074 | 0,065 | 0,267 | 0,245 | 0,109 | 0,276 | 0,311 | 0,710 |
| 0,78 | -0,005 | 0,207 | 0,311 | 0,482 | -0,008 | 0,077 | 0,296 | 0,309 | 0,078 | 0,215 | 0,315 | 0,680 |
| 0,79 | 0,058 | 0,235 | 0,344 | 0,495 | -0,010 | 0,097 | 0,318 | 0,326 | 0,099 | 0,196 | 0,396 | 0,710 |
| 0,80 | 0,083 | 0,247 | 0,395 | 0,558 | 0,049 | 0,158 | 0,369 | 0,288 | 0,106 | 0,216 | 0,467 | 0,770 |
| 0,81 | 0,126 | 0,304 | 0,426 | 0,628 | 0,065 | 0,245 | 0,408 | 0,350 | 0,114 | 0,379 | 0,549 | 0,824 |
| 0,82 | 0,104 | 0,263 | 0,439 | 0,605 | 0,085 | 0,258 | 0,434 | 0,404 | 0,212 | 0,410 | 0,595 | 0,879 |
| 0,83 | 0,132 | 0,310 | 0,473 | 0,649 | 0,035 | 0,229 | 0,430 | 0,399 | 0,259 | 0,360 | 0,652 | 0,861 |
| 0,84 | 0,172 | 0,328 | 0,552 | 0,689 | 0,128 | 0,324 | 0,590 | 0,465 | 0,265 | 0,261 | 0,644 | 0,881 |
| 0,85 | 0,294 | 0,450 | 0,582 | 0,772 | 0,148 | 0,341 | 0,588 | 0,474 | 0,241 | 0,311 | 0,639 | 0,891 |
| 0,86 | 0,275 | 0,397 | 0,586 | 0,799 | 0,160 | 0,454 | 0,594 | 0,586 | 0,195 | 0,402 | 0,593 | 0,976 |
| 0,87 | 0,261 | 0,377 | 0,580 | 0,861 | 0,235 | 0,499 | 0,665 | 0,590 | 0,230 | 0,337 | 0,609 | 1,087 |
| 0,88 | 0,203 | 0,350 | 0,548 | 0,819 | 0,389 | 0,470 | 0,734 | 0,688 | 0,185 | 0,393 | 0,632 | 1,102 |
| 0,89 | 0,144 | 0,409 | 0,519 | 0,889 | 0,441 | 0,590 | 0,684 | 0,717 | 0,201 | 0,404 | 0,671 | 1,099 |
| 0,90 | 0,259 | 0,530 | 0,670 | 1,008 | 0,320 | 0,536 | 0,531 | 0,828 | 0,190 | 0,514 | 0,649 | 1,104 |
| 0,91 | 0,340 | 0,554 | 0,684 | 1,044 | 0,242 | 0,449 | 0,531 | 0,863 | 0,218 | 0,462 | 0,639 | 1,333 |
| 0,92 | 0,209 | 0,613 | 0,620 | 1,056 | 0,070 | 0,538 | 0,496 | 0,826 | 0,409 | 1,028 | 0,950 | 1,586 |
| 0,93 | 0,324 | 0,506 | 0,583 | 1,286 | 0,459 | 0,711 | 0,718 | 0,975 | 0,630 | 0,688 | 1,196 | 1,882 |
| 0,94 | 0,395 | 0,616 | 0,934 | 1,655 | 0,518 | 1,032 | 0,757 | 1,049 | 0,500 | 0,403 | 1,080 | 1,691 |
| 0,95 | 0,415 | 0,516 | 0,925 | 1,672 | 0,474 | 0,928 | 0,714 | 1,345 | 0,157 | 0,112 | 0,860 | 1,506 |
| 0,96 | 0,222 | 0,307 | 0,717 | 1,649 | 0,376 | 0,600 | 0,759 | 1,675 | 0,478 | 0,175 | 0,710 | 1,496 |
| 0,97 | 0,434 | 0,399 | 1,029 | 1,664 | 0,425 | 0,811 | 0,730 | 1,970 | 0,859 | 0,381 | 1,323 | 1,701 |
| 0,98 | 0,636 | 0,124 | 1,335 | 1,978 | 0,422 | 0,635 | 1,364 | 2,247 | 0,495 | -0,079 | 1,900 | 1,819 |
| 0,99 | -0,711 | -0,925 | 1,217 | 2,317 | -0,194 | -0,210 | 1,471 | 3,692 | -0,102 | -1,599 | 1,722 | 1,236 |

Table S4: **Quantile regression coefficients for men, stratified by low and high educational attainment, calculated biennially using variable reference time periods (period-to-period analysis).**

| **Men** | | | | | **High education** | | | | **Low education** | | | |
| --- | --- | --- | --- | --- | --- | --- | --- | --- | --- | --- | --- | --- |
| **Quantile** | **Estimates** | | | | | | | | | | | |
|  | 2010-13 | 2012-15 | 2014-17 | 2016-19 | 2010-13 | 2012-15 | 2014-17 | 2016-19 | 2010-13 | 2012-15 | 2014-17 | 2016-19 |
| 0,01 | 0,037 | -0,555 | 0,464 | -0,388 | -0,155 | -0,700 | 1,029 | -0,751 | 0,166 | -0,222 | -0,481 | 0,296 |
| 0,02 | -0,069 | -0,145 | 0,135 | -0,206 | 0,020 | -0,667 | 0,760 | -0,324 | -0,108 | 0,105 | -0,383 | 0,031 |
| 0,03 | -0,002 | -0,012 | 0,006 | -0,189 | -0,029 | -0,285 | 0,376 | -0,318 | -0,072 | 0,105 | -0,118 | 0,069 |
| 0,04 | -0,116 | -0,007 | 0,070 | -0,189 | 0,019 | -0,150 | 0,241 | -0,406 | -0,206 | 0,005 | 0,020 | 0,066 |
| 0,05 | -0,019 | -0,047 | 0,022 | -0,075 | -0,080 | -0,059 | 0,160 | -0,371 | -0,104 | 0,044 | -0,119 | 0,120 |
| 0,06 | 0,023 | -0,098 | 0,069 | -0,056 | 0,065 | -0,114 | 0,194 | -0,265 | -0,138 | 0,034 | -0,075 | 0,144 |
| 0,07 | 0,026 | -0,064 | 0,046 | -0,064 | 0,105 | -0,192 | 0,192 | -0,170 | -0,026 | 0,064 | -0,166 | 0,136 |
| 0,08 | 0,067 | -0,050 | -0,012 | -0,045 | 0,125 | -0,113 | 0,089 | -0,189 | 0,025 | 0,040 | -0,191 | 0,239 |
| 0,09 | 0,036 | 0,020 | -0,041 | -0,056 | 0,114 | -0,109 | 0,115 | -0,194 | -0,034 | 0,146 | -0,197 | 0,245 |
| 0,1 | 0,020 | 0,028 | -0,031 | -0,042 | 0,074 | -0,054 | 0,107 | -0,215 | 0,000 | 0,087 | -0,125 | 0,196 |
| 0,11 | 0,012 | 0,038 | -0,018 | -0,090 | 0,089 | -0,030 | 0,121 | -0,249 | -0,024 | 0,122 | -0,187 | 0,187 |
| 0,12 | 0,001 | 0,045 | -0,019 | -0,080 | 0,065 | -0,021 | 0,102 | -0,220 | -0,025 | 0,118 | -0,123 | 0,170 |
| 0,13 | 0,010 | 0,024 | 0,009 | -0,111 | 0,051 | -0,020 | 0,104 | -0,281 | -0,101 | 0,135 | -0,076 | 0,214 |
| 0,14 | 0,027 | 0,036 | 0,012 | -0,136 | 0,044 | -0,035 | 0,138 | -0,318 | -0,158 | 0,191 | -0,105 | 0,189 |
| 0,15 | 0,023 | 0,016 | 0,051 | -0,124 | 0,068 | -0,078 | 0,148 | -0,355 | -0,204 | 0,246 | -0,096 | 0,138 |
| 0,16 | -0,034 | 0,032 | 0,069 | -0,124 | 0,106 | -0,064 | 0,080 | -0,319 | -0,206 | 0,223 | -0,071 | 0,151 |
| 0,17 | -0,045 | 0,088 | 0,031 | -0,075 | 0,087 | -0,069 | 0,145 | -0,355 | -0,124 | 0,197 | -0,051 | 0,091 |
| 0,18 | -0,054 | 0,091 | 0,031 | -0,081 | 0,069 | -0,076 | 0,177 | -0,367 | -0,134 | 0,169 | 0,019 | 0,004 |
| 0,19 | -0,062 | 0,060 | 0,072 | -0,118 | 0,080 | -0,068 | 0,173 | -0,354 | -0,105 | 0,203 | -0,046 | 0,002 |
| 0,2 | 0,000 | 0,029 | 0,110 | -0,160 | 0,082 | -0,039 | 0,154 | -0,313 | -0,096 | 0,184 | -0,024 | -0,020 |
| 0,21 | -0,006 | 0,039 | 0,106 | -0,138 | 0,056 | -0,021 | 0,125 | -0,266 | -0,099 | 0,191 | -0,010 | -0,009 |
| 0,22 | -0,015 | 0,100 | 0,037 | -0,109 | 0,020 | -0,028 | 0,161 | -0,284 | -0,065 | 0,164 | -0,006 | -0,011 |
| 0,23 | -0,011 | 0,093 | 0,035 | -0,125 | 0,054 | -0,060 | 0,211 | -0,311 | -0,060 | 0,151 | -0,021 | 0,076 |
| 0,24 | 0,051 | 0,046 | 0,033 | -0,130 | 0,047 | -0,038 | 0,195 | -0,300 | -0,031 | 0,136 | 0,004 | 0,055 |
| 0,25 | 0,026 | 0,079 | 0,012 | -0,118 | 0,069 | -0,035 | 0,154 | -0,279 | -0,040 | 0,131 | 0,010 | 0,047 |
| 0,26 | 0,030 | 0,063 | 0,011 | -0,084 | 0,116 | 0,006 | 0,075 | -0,213 | -0,036 | 0,156 | -0,025 | 0,097 |
| 0,27 | 0,022 | 0,039 | 0,011 | -0,048 | 0,144 | -0,045 | 0,089 | -0,205 | -0,020 | 0,105 | 0,005 | 0,115 |
| 0,28 | 0,032 | 0,043 | 0,018 | -0,030 | 0,134 | -0,008 | 0,064 | -0,202 | 0,006 | 0,076 | -0,012 | 0,135 |
| 0,29 | 0,032 | 0,039 | 0,027 | -0,029 | 0,151 | 0,004 | 0,039 | -0,186 | 0,013 | 0,060 | 0,006 | 0,116 |
| 0,3 | 0,030 | 0,019 | 0,038 | -0,034 | 0,136 | -0,021 | 0,020 | -0,150 | 0,026 | 0,063 | 0,003 | 0,161 |
| 0,31 | 0,038 | 0,011 | 0,031 | 0,000 | 0,099 | -0,025 | 0,025 | -0,107 | 0,015 | 0,056 | -0,007 | 0,209 |
| 0,32 | 0,051 | -0,019 | 0,050 | 0,024 | 0,105 | -0,055 | 0,054 | -0,100 | -0,005 | 0,069 | 0,012 | 0,180 |
| 0,33 | 0,041 | -0,023 | 0,039 | 0,052 | 0,081 | -0,002 | 0,024 | -0,105 | -0,006 | 0,040 | 0,088 | 0,190 |
| 0,34 | 0,038 | -0,006 | 0,036 | 0,051 | 0,051 | -0,020 | 0,041 | -0,089 | 0,012 | 0,054 | 0,056 | 0,165 |
| 0,35 | 0,025 | -0,007 | 0,026 | 0,095 | 0,069 | -0,072 | 0,079 | -0,084 | 0,008 | 0,068 | 0,064 | 0,174 |
| 0,36 | 0,026 | -0,012 | 0,036 | 0,089 | 0,072 | -0,076 | 0,088 | -0,093 | 0,016 | 0,037 | 0,059 | 0,179 |
| 0,37 | 0,019 | 0,003 | 0,040 | 0,087 | 0,094 | -0,112 | 0,119 | -0,065 | 0,073 | 0,002 | 0,032 | 0,170 |
| 0,38 | 0,042 | -0,007 | 0,071 | 0,055 | 0,044 | -0,085 | 0,096 | -0,029 | 0,060 | 0,022 | 0,045 | 0,154 |
| 0,39 | 0,040 | -0,014 | 0,074 | 0,080 | 0,031 | -0,076 | 0,095 | -0,009 | 0,062 | 0,004 | 0,085 | 0,116 |
| 0,4 | 0,030 | -0,010 | 0,071 | 0,074 | 0,040 | -0,053 | 0,056 | 0,030 | 0,086 | 0,006 | 0,083 | 0,109 |
| 0,41 | 0,052 | -0,030 | 0,065 | 0,108 | 0,057 | -0,093 | 0,084 | 0,025 | 0,093 | -0,002 | 0,082 | 0,154 |
| 0,42 | 0,062 | -0,034 | 0,069 | 0,096 | 0,062 | -0,060 | 0,049 | 0,005 | 0,104 | 0,013 | 0,058 | 0,146 |
| 0,43 | 0,054 | -0,012 | 0,064 | 0,082 | 0,097 | -0,056 | 0,067 | 0,010 | 0,105 | 0,020 | 0,048 | 0,151 |
| 0,44 | 0,039 | -0,006 | 0,075 | 0,071 | 0,111 | -0,070 | 0,059 | 0,025 | 0,106 | 0,063 | 0,000 | 0,173 |
| 0,45 | 0,044 | 0,002 | 0,083 | 0,048 | 0,061 | -0,074 | 0,094 | 0,000 | 0,125 | 0,045 | -0,011 | 0,182 |
| 0,46 | 0,044 | -0,004 | 0,084 | 0,058 | 0,051 | -0,050 | 0,079 | 0,002 | 0,115 | -0,001 | 0,044 | 0,187 |
| 0,47 | 0,052 | 0,005 | 0,066 | 0,078 | 0,058 | -0,059 | 0,079 | 0,018 | 0,133 | 0,020 | 0,056 | 0,146 |
| 0,48 | 0,063 | -0,021 | 0,062 | 0,096 | 0,051 | -0,052 | 0,089 | 0,026 | 0,130 | 0,080 | 0,001 | 0,176 |
| 0,49 | 0,056 | 0,009 | 0,026 | 0,082 | 0,022 | -0,030 | 0,098 | 0,005 | 0,107 | 0,058 | 0,045 | 0,157 |
| 0,5 | 0,068 | -0,013 | 0,030 | 0,104 | -0,001 | -0,022 | 0,092 | 0,008 | 0,147 | 0,026 | 0,107 | 0,105 |
| 0,51 | 0,069 | -0,018 | 0,034 | 0,116 | -0,010 | -0,007 | 0,101 | -0,021 | 0,170 | -0,041 | 0,137 | 0,096 |
| 0,52 | 0,090 | -0,026 | 0,063 | 0,096 | 0,000 | 0,000 | 0,098 | 0,005 | 0,205 | -0,048 | 0,179 | 0,080 |
| 0,53 | 0,073 | 0,019 | 0,034 | 0,096 | -0,004 | 0,000 | 0,070 | 0,024 | 0,186 | -0,056 | 0,239 | 0,013 |
| 0,54 | 0,085 | 0,022 | 0,016 | 0,129 | 0,014 | -0,031 | 0,054 | 0,039 | 0,217 | -0,039 | 0,201 | 0,035 |
| 0,55 | 0,093 | 0,021 | 0,051 | 0,110 | 0,021 | -0,041 | 0,079 | 0,009 | 0,205 | -0,043 | 0,196 | 0,089 |
| 0,56 | 0,125 | -0,033 | 0,082 | 0,120 | 0,029 | -0,051 | 0,074 | 0,041 | 0,205 | -0,067 | 0,210 | 0,139 |
| 0,57 | 0,132 | -0,044 | 0,111 | 0,093 | 0,048 | -0,062 | 0,066 | 0,034 | 0,156 | -0,011 | 0,165 | 0,121 |
| 0,58 | 0,133 | -0,058 | 0,140 | 0,080 | 0,034 | -0,059 | 0,075 | 0,052 | 0,143 | 0,028 | 0,188 | 0,092 |
| 0,59 | 0,119 | -0,053 | 0,168 | 0,058 | 0,054 | -0,042 | 0,064 | 0,040 | 0,114 | 0,032 | 0,169 | 0,084 |
| 0,6 | 0,059 | -0,011 | 0,141 | 0,070 | 0,061 | -0,036 | 0,028 | 0,092 | 0,081 | 0,066 | 0,117 | 0,099 |
| 0,61 | 0,064 | -0,012 | 0,159 | 0,073 | 0,092 | -0,005 | 0,027 | 0,106 | 0,078 | 0,089 | 0,097 | 0,115 |
| 0,62 | 0,065 | 0,017 | 0,115 | 0,122 | 0,086 | -0,019 | 0,033 | 0,145 | 0,044 | 0,094 | 0,094 | 0,101 |
| 0,63 | 0,024 | 0,043 | 0,138 | 0,069 | 0,036 | -0,025 | 0,050 | 0,151 | 0,040 | 0,104 | 0,146 | 0,034 |
| 0,64 | 0,008 | 0,048 | 0,163 | 0,084 | -0,015 | -0,055 | 0,084 | 0,150 | 0,030 | 0,113 | 0,139 | 0,058 |
| 0,65 | 0,028 | 0,075 | 0,100 | 0,088 | -0,030 | -0,029 | 0,079 | 0,130 | 0,029 | 0,094 | 0,134 | 0,166 |
| 0,66 | 0,024 | 0,080 | 0,103 | 0,070 | -0,010 | 0,017 | 0,096 | 0,087 | -0,009 | 0,121 | 0,154 | 0,137 |
| 0,67 | 0,006 | 0,095 | 0,103 | 0,068 | -0,020 | 0,026 | 0,113 | 0,084 | 0,005 | 0,090 | 0,163 | 0,131 |
| 0,68 | -0,018 | 0,117 | 0,114 | 0,033 | -0,032 | 0,061 | 0,091 | 0,079 | -0,005 | 0,095 | 0,149 | 0,200 |
| 0,69 | 0,001 | 0,128 | 0,119 | 0,032 | -0,071 | 0,073 | 0,135 | 0,037 | 0,002 | 0,130 | 0,127 | 0,235 |
| 0,7 | 0,010 | 0,122 | 0,123 | 0,076 | -0,069 | 0,097 | 0,153 | 0,044 | -0,010 | 0,155 | 0,145 | 0,182 |
| 0,71 | -0,030 | 0,111 | 0,154 | 0,074 | -0,010 | 0,061 | 0,133 | 0,064 | -0,030 | 0,172 | 0,140 | 0,162 |
| 0,72 | -0,035 | 0,098 | 0,175 | 0,073 | -0,032 | 0,131 | 0,120 | 0,018 | -0,049 | 0,212 | 0,069 | 0,226 |
| 0,73 | -0,016 | 0,090 | 0,192 | 0,098 | -0,033 | 0,138 | 0,088 | 0,051 | -0,051 | 0,240 | 0,064 | 0,321 |
| 0,74 | -0,032 | 0,111 | 0,173 | 0,103 | -0,005 | 0,136 | 0,107 | 0,050 | -0,001 | 0,217 | 0,064 | 0,339 |
| 0,75 | -0,015 | 0,123 | 0,181 | 0,079 | -0,025 | 0,159 | 0,132 | 0,009 | 0,025 | 0,156 | 0,095 | 0,392 |
| 0,76 | 0,004 | 0,173 | 0,144 | 0,052 | -0,074 | 0,167 | 0,162 | -0,001 | 0,118 | 0,106 | 0,018 | 0,460 |
| 0,77 | 0,025 | 0,181 | 0,126 | 0,100 | -0,074 | 0,139 | 0,202 | -0,022 | 0,109 | 0,168 | 0,035 | 0,399 |
| 0,78 | -0,005 | 0,213 | 0,104 | 0,171 | -0,008 | 0,085 | 0,219 | 0,012 | 0,078 | 0,137 | 0,100 | 0,365 |
| 0,79 | 0,058 | 0,177 | 0,109 | 0,151 | -0,010 | 0,107 | 0,221 | 0,008 | 0,099 | 0,097 | 0,200 | 0,314 |
| 0,8 | 0,083 | 0,165 | 0,148 | 0,163 | 0,049 | 0,101 | 0,211 | -0,081 | 0,106 | 0,105 | 0,244 | 0,302 |
| 0,81 | 0,126 | 0,177 | 0,123 | 0,201 | 0,065 | 0,180 | 0,162 | -0,058 | 0,114 | 0,265 | 0,170 | 0,275 |
| 0,82 | 0,104 | 0,159 | 0,176 | 0,166 | 0,085 | 0,173 | 0,176 | -0,030 | 0,212 | 0,198 | 0,185 | 0,284 |
| 0,83 | 0,132 | 0,177 | 0,163 | 0,175 | 0,035 | 0,194 | 0,201 | -0,031 | 0,259 | 0,101 | 0,293 | 0,209 |
| 0,84 | 0,172 | 0,156 | 0,224 | 0,136 | 0,128 | 0,196 | 0,266 | -0,125 | 0,265 | 0,020 | 0,359 | 0,238 |
| 0,85 | 0,294 | 0,156 | 0,133 | 0,190 | 0,148 | 0,194 | 0,246 | -0,114 | 0,241 | 0,070 | 0,327 | 0,252 |
| 0,86 | 0,275 | 0,122 | 0,189 | 0,213 | 0,160 | 0,294 | 0,140 | -0,008 | 0,195 | 0,207 | 0,192 | 0,383 |
| 0,87 | 0,261 | 0,116 | 0,203 | 0,281 | 0,235 | 0,264 | 0,166 | -0,075 | 0,230 | 0,107 | 0,271 | 0,479 |
| 0,88 | 0,203 | 0,147 | 0,198 | 0,271 | 0,389 | 0,081 | 0,264 | -0,046 | 0,185 | 0,208 | 0,233 | 0,470 |
| 0,89 | 0,144 | 0,265 | 0,110 | 0,370 | 0,441 | 0,149 | 0,094 | 0,033 | 0,201 | 0,203 | 0,267 | 0,427 |
| 0,9 | 0,259 | 0,263 | 0,140 | 0,338 | 0,320 | 0,216 | -0,005 | 0,296 | 0,190 | 0,324 | 0,135 | 0,455 |
| 0,91 | 0,340 | 0,214 | 0,130 | 0,360 | 0,242 | 0,206 | 0,083 | 0,331 | 0,218 | 0,245 | 0,176 | 0,694 |
| 0,92 | 0,209 | 0,404 | 0,007 | 0,436 | 0,070 | 0,468 | -0,041 | 0,330 | 0,409 | 0,084 | 0,433 | 0,636 |
| 0,93 | 0,324 | 0,182 | 0,077 | 0,703 | 0,459 | 0,252 | 0,006 | 0,258 | 0,630 | 0,058 | 0,509 | 0,686 |
| 0,94 | 0,395 | 0,221 | 0,318 | 0,721 | 0,518 | 0,514 | -0,274 | 0,291 | 0,500 | -0,097 | 0,677 | 0,611 |
| 0,95 | 0,415 | 0,101 | 0,409 | 0,747 | 0,474 | 0,455 | -0,215 | 0,631 | 0,157 | -0,045 | 0,748 | 0,646 |
| 0,96 | 0,222 | 0,085 | 0,410 | 0,931 | 0,376 | 0,224 | 0,159 | 0,916 | 0,478 | -0,275 | 0,508 | 0,786 |
| 0,97 | 0,434 | -0,035 | 0,631 | 0,634 | 0,425 | 0,386 | -0,082 | 1,240 | 0,859 | -0,478 | 0,942 | 0,379 |
| 0,98 | 0,636 | -0,512 | 1,211 | 0,643 | 0,422 | 0,213 | 0,728 | 0,884 | 0,495 | -0,574 | 1,979 | -0,081 |
| 0,99 | -0,711 | -0,214 | 2,143 | 1,100 | -0,194 | -0,016 | 1,681 | 2,221 | -0,102 | -1,497 | 3,321 | -0,485 |


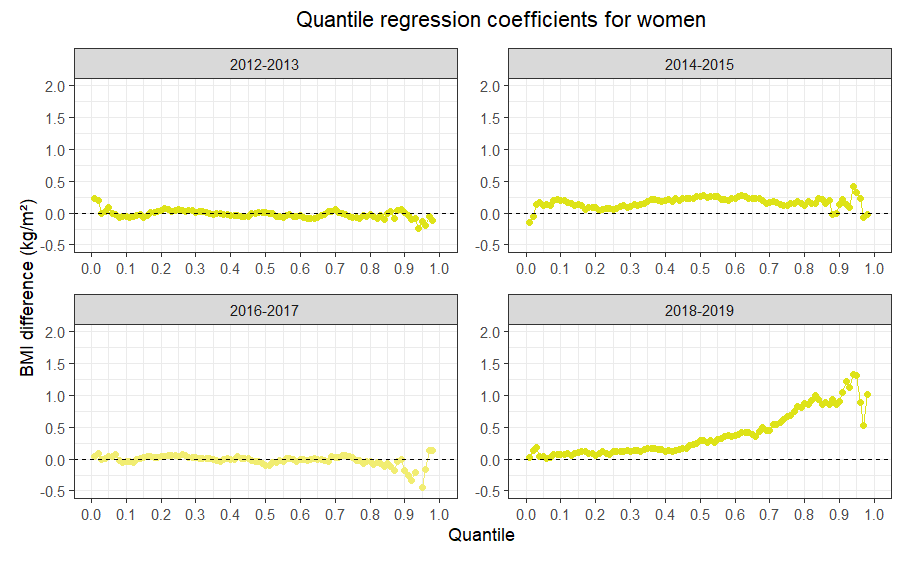


Figure S1: BMI differences for the 0.01 to 0.98 quantiles of the BMI distribution for women. The horizontal line at 0.0 is the line of equality and a point on this line means that there is no difference in the predicted BMI at that specific quantile compared to the previous time period. The left upper box shows 2012/13 against 2010/11.


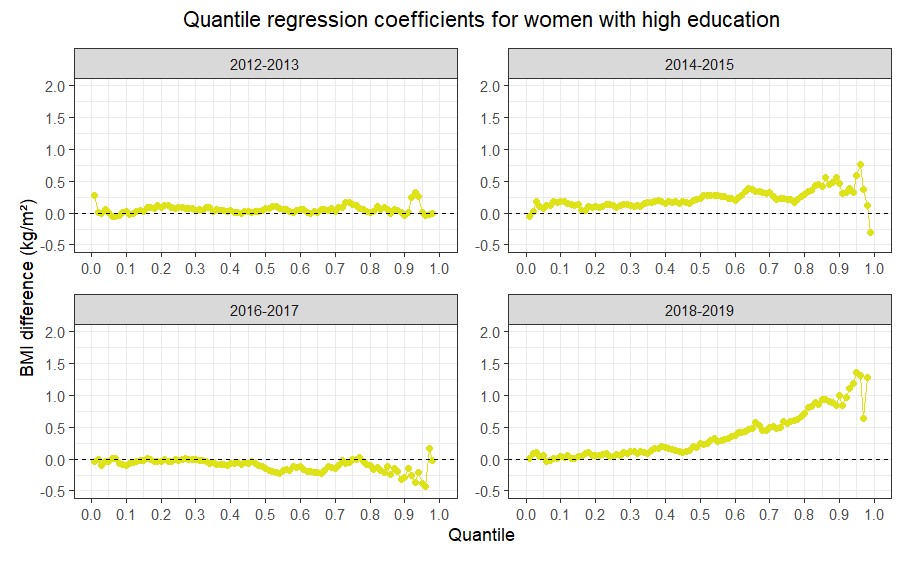


Figure S2: BMI differences for the 0.01 to 0.98 quantiles of the BMI distribution for women with high education (at least post-secondary education). The left lower box shows 2016/17 against 2014/15.


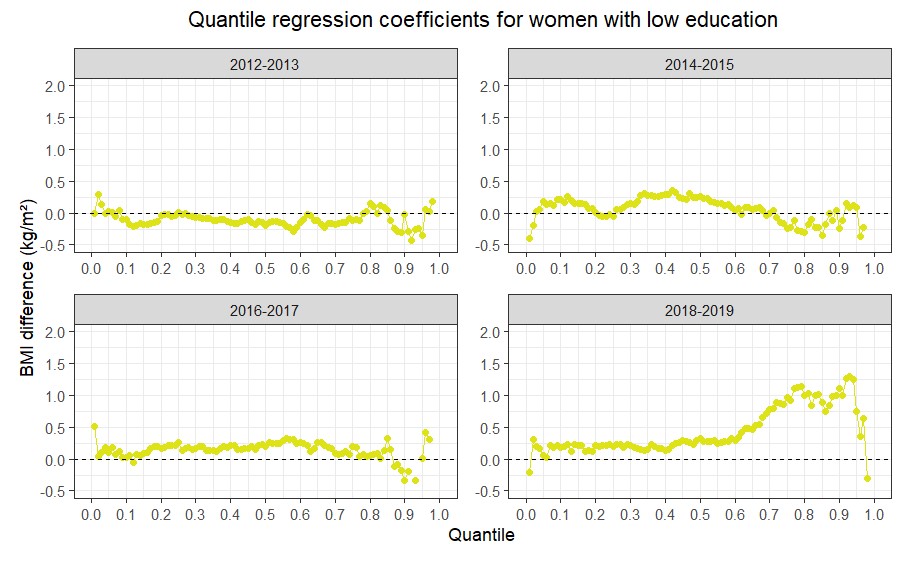


Figure S3: BMI differences for the 0.01 to 0.98 quantiles of the BMI distribution for women with low education. The right lower box shows 2018/19 against 2016/17.


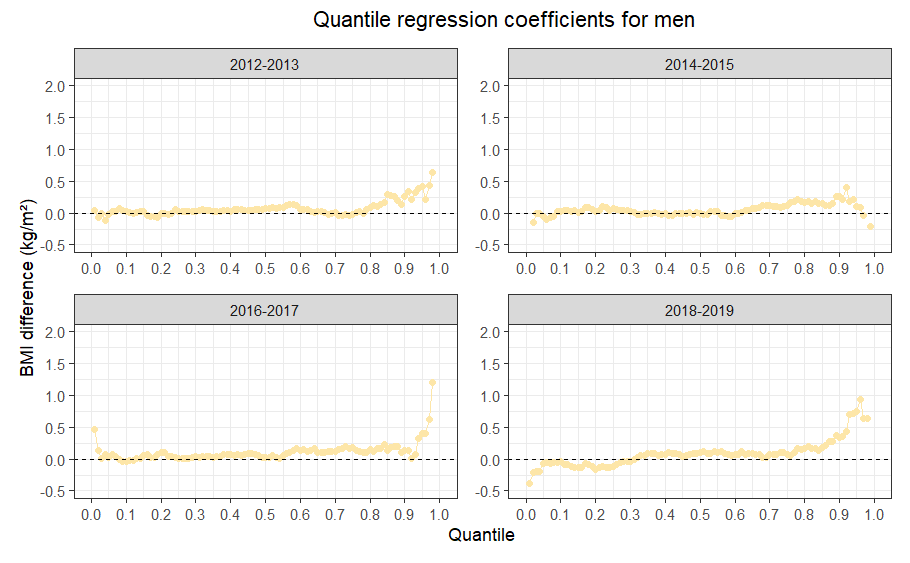


Figure S4: BMI differences for the 0.01 to 0.98 quantiles of the BMI distribution for men. The horizontal line at 0.0 is the line of equality and a point on this line means that there is no difference in the predicted BMI at that specific quantile compared to the previous time period. The left upper box shows 2012/13 against 2010/11.


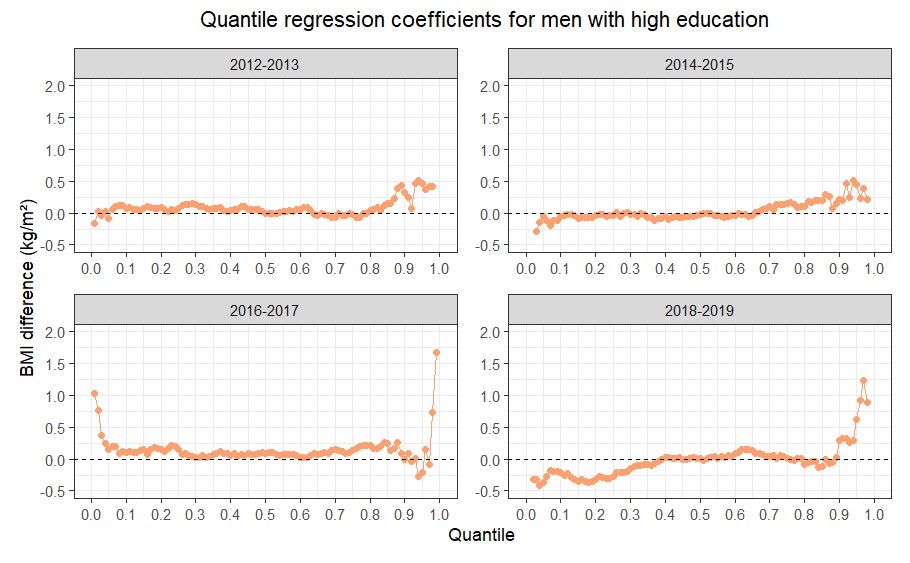


Figure S5: BMI differences for the 0.01 to 0.98 quantiles of the BMI distribution for men with high education. The left lower box shows 2016/17 against 2014/15.


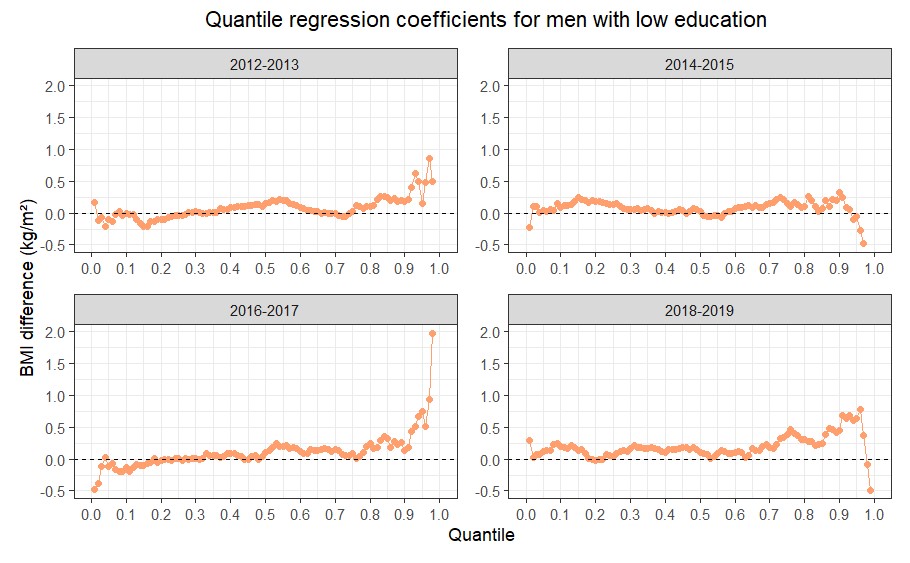


Figure S6: BMI differences for the 0.01 to 0.98 quantiles of the BMI distribution for men with low education. The right lower box shows 2018/19 against 2016/17.
